# Comparative analysis of cell lineage differentiation during hepatogenesis in humans and mice at the single-cell transcriptome level

**DOI:** 10.1038/s41422-020-0378-6

**Published:** 2020-07-20

**Authors:** Xin Wang, Li Yang, Yan-Chun Wang, Zi-Ran Xu, Ye Feng, Jing Zhang, Yi Wang, Cheng-Ran Xu

**Affiliations:** 1 Ministry of Education Key Laboratory of Cell Proliferation and Differentiation, College of Life Sciences, Department of Human Anatomy, Histology, and Embryology, and School of Basic Medical Sciences, Peking University Health Science Center, Peking University, Beijing, 100871 China; 2 Haidian Maternal & Child Health Hospital, Beijing, 100080 China

**Keywords:** Developmental biology, Stem-cell differentiation, Stem-cell differentiation, Developmental biology

## Abstract

During embryogenesis, the liver is the site of hepatogenesis and hematopoiesis and contains many cell lineages derived from the endoderm and mesoderm. However, the characteristics and developmental programs of many of these cell lineages remain unclear, especially in humans. Here, we performed single-cell RNA sequencing of whole human and mouse fetal livers throughout development. We identified four cell lineage families of endoderm-derived, erythroid, non-erythroid hematopoietic, and mesoderm-derived non-hematopoietic cells, and defined the developmental pathways of the major cell lineage families. In both humans and mice, we identified novel markers of hepatic lineages and an ID3^+^ subpopulation of hepatoblasts as well as verified that hepatoblast differentiation follows the “default-directed” model. Additionally, we found that human but not mouse fetal hepatocytes display heterogeneity associated with expression of metabolism-related genes. We described the developmental process of erythroid progenitor cells during human and mouse hematopoiesis. Moreover, despite the general conservation of cell differentiation programs between species, we observed different cell lineage compositions during hematopoiesis in the human and mouse fetal livers. Taken together, these results reveal the dynamic cell landscape of fetal liver development and illustrate the similarities and differences in liver development between species, providing an extensive resource for inducing various liver cell lineages in vitro.

## Introduction

The liver consists of greater than 20 cell types, including hepatocytes, biliary ductal cells (cholangiocytes), liver endothelial cells, hepatic stellate cells, Kupffer cells, mesothelial cells, and various circulatory immune cells, which are all organized to form the foundation for liver functions, including nutrient metabolism, drug detoxification, and immune responses.^1–4^ Cell lineage differentiation and organogenesis mainly occur in the fetal stage. Although the development of certain cell lineages across fetal development in the mouse liver has been studied,^5–8^ the cell type components and their development pathways during embryogenesis have not been comprehensively defined, especially in humans. In addition, a comparative study of fetal liver development between two important species, mice and humans, is also critical to our understanding of the mechanisms of liver development and regeneration, but we know little about the degree to which liver development is conserved between humans and mice.

During embryogenesis, hepatocytes and cholangiocytes, two major lineages for the majority of liver functions, are derived from bipotential hepatoblasts, which are widely considered to originate from the definitive endoderm at 3–4 weeks post-coitum (W) in humans or at embryonic day (E) 8.5–E9.0 in mice.^9^ Several transcription factors (TFs), such as FOXA1/2, GATA4/6, HHEX, and HNF1A/1B, are involved in hepatoblast specification.^9,10^ The differentiation of hepatoblasts into hepatocytes and cholangiocytes begins at E13.5 in mice, and characteristic differentiated hepatobiliary cells can be clearly observed at approximately W7 in humans.^5,11^ Several single-cell RNA sequencing (scRNA-seq) studies have investigated the developmental processes that underlie hepatic lineage development.^5,12–14^ Our previous scRNA-seq analysis revealed a “default-directed” model of hepatoblast differentiation in mice.^5^ In this model, hepatoblasts that have begun to express hepatocyte function-related genes choose the default fate of becoming hepatocytes, while cholangiocyte differentiation involves a sharp detour from this default path via de novo activation of additional TFs and multiple signaling pathways, including the Sox4, Sox9, Hnf1b and Notch, Tgfb, Wnt, FGF, Hippo-Yap, and MAPK pathways.^5,15–22^ However, whether this “default-directed” regulatory strategy is conserved in humans remains unknown.

In the processes of hepatoblast proliferation and liver bud formation, surrounding mesoderm-derived cells invade the developing liver tissue and further differentiate into various vessels and mesenchymal cells, the latter producing hepatic stellate cells and mesothelial cells.^23^ These mesoderm-derived cells provide critical signals to regulate hepatobiliary development and contribute to the formation of the liver structure.^3^ However, the cell fate of these mesoderm-derived cells in human fetal liver is largely unknown.

The fetal liver is a fundamental organ for hematopoiesis during embryogenesis.^24^ Hematopoietic stem and progenitor cells (HSPCs) that originate from the yolk sac contribute to the first “wave” of hematopoiesis by producing cells with limited hematopoietic activities, such as circulating primitive erythrocytes, megakaryocytes, macrophages, and some granulocytes.^25–27^ HSPCs derived from the aorta-gonad-mesonephros (AGM) region evoke another “wave” of hematopoiesis, circulate in blood vessels, and seed the fetal liver.^28,29^ Immigration of HSPCs into the liver bud occurs at approximately W6 in humans or E12.0 in mice.^24,28,30^ Then, the fetal liver becomes the major hematopoietic organ and provides a specific niche for HSPC proliferation and differentiation.^29,30^ HSPCs give rise to three main lineages of blood cells in fetal liver: megakaryocyte–erythroid–mast cells, lymphoid cells, and myeloid cells.^31^ Hematopoiesis also promotes the morphological and functional maturation of hepatocytes in fetal liver.^32^ As the liver matures, HSPCs migrate out of the liver and permanently reside in the bone marrow. Using scRNA-seq, several recent studies have revealed precise models of hematopoiesis in the human fetal liver.^31,33–35^ However, whether the fetal liver hematopoiesis model is conserved across species remains unclear.

In this study, we performed unbiased scRNA-seq of fetal livers over developmental time from W5 to W19 in humans and from E11.0 to E17.5 in mice. We systematically identified four major cell lineage families: endoderm-derived lineages, erythroid lineages, non-erythroid hematopoietic lineages, and mesoderm-derived non-hematopoietic lineages, as well as various specific cell types within each family in humans and mice. We also defined cell lineage differentiation pathways in the human and mouse fetal livers. In addition, we observed significant differences in gene expression, cell composition, cell heterogeneity, and lineage differentiation pathways during mouse and human fetal liver development.

## Results

### Cell population landscapes in the human and mouse fetal livers

We collected fetal livers at 9 time points from W5 to W19 in humans and 6 time points from E11.0 to E17.5 in mice. At W5–W6 in humans and E11.0–E11.5 in mice, small clusters of red blood cells were scattered throughout the liver (Supplementary information, Fig. S1a). At W7 in humans and E13.0 in mice, the livers were filled with red blood cells and were completely reddened (Supplementary information, Fig. S1a). This dramatic morphological change indicated that both human and mouse fetal livers experienced blood immigration and rapid growth during the period studied in our experiments.

**Fig. 1:**
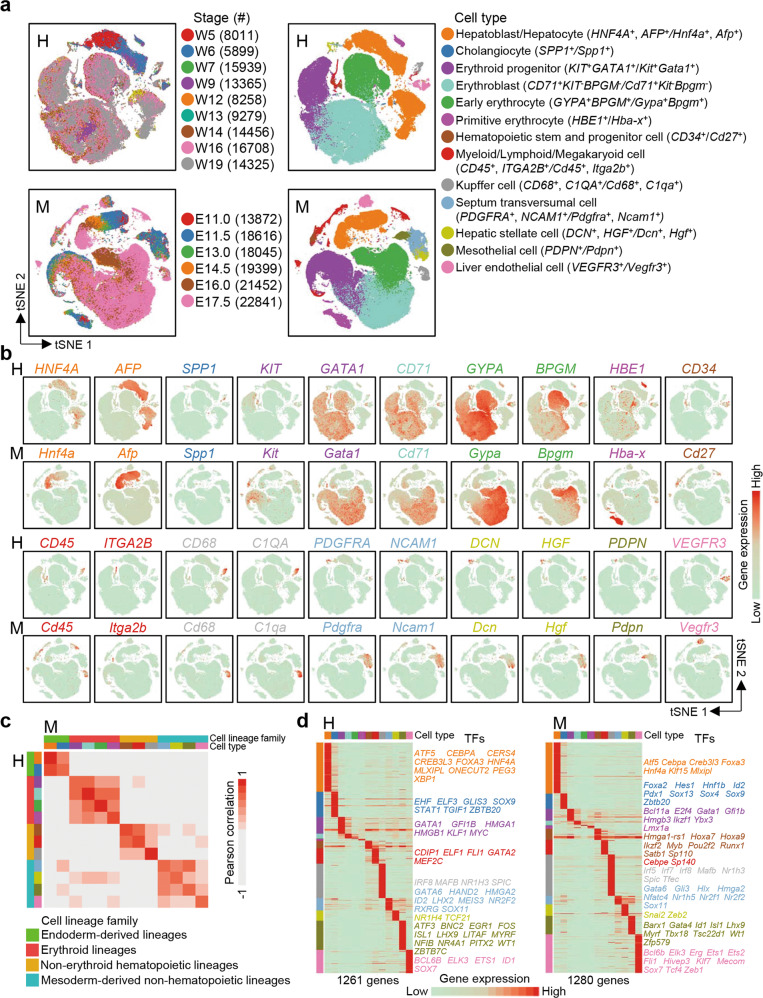
scRNA-seq identified major cell types and cell lineage families in the human and mouse fetal livers. **a** t-SNE plots showing the developmental stages (left) and cell clusters (right) of human (H) and mouse (M) fetal liver development. **b** t-SNE plots showing the expression levels of marker genes for each cell population. **c** Heatmaps showing the Pearson correlations of 13 major cell types between human (H) and mouse (M). **d** Differentially expressed genes in human (H) and mouse (M) cell populations. Each column represents a cell type and each row represents a gene. The TFs associated with each cell type are listed on the right. The color scheme is the same as **a**.

After digestion of isolated fetal livers, we performed unbiased scRNA-seq using the 10× Genomics platform and obtained a total of > 100,000 single-cell transcriptomes for each species (Supplementary information, Fig. S1b); we detected an average of ~2000 genes in non-erythrocytes and 1000 genes in erythrocytes (*HBA1*^+^, *HBE1*^+^/*Hba-a1*^+^, *Hba-x*^+^) in the human and mouse samples (Supplementary information, Fig. S1c). This result is consistent with the finding that RNA synthesis declines upon erythrocyte maturation.^36^ In this study, we used the same software and strategies to analyze the human and mouse datasets. After dimension reduction, doublet removal (Supplementary information, Fig. S1d), iterative clustering, and batch effect correction, we identified 13 major cell types across all developmental stages in both the human and mouse fetal livers based on marker gene expression (Fig. 1a; Supplementary information, Table S1). These cell types included hepatoblasts/hepatocytes (*HNF4A*^*+*^, *AFP*^*+*^/*Hnf4a*^*+*^, *Afp*^*+*^), cholangiocytes (*SPP1*^*+*^/*Spp1*^*+*^), erythroid progenitors (*KIT*^*+*^*GATA1*^*+*^/*Kit*^*+*^*Gata1*^*+*^), erythroblasts (*CD71*^*+*^*KIT*^*–*^*BPGM*^*–*^/*Cd71*^*+*^*Kit*^*–*^*Bpgm*^*–*^), early erythrocytes (*GYPA*^+^*BPGM*^*+*^/*Gypa*^+^*Bpgm*^*+*^),^37,38^ primitive erythrocytes (*HBE1*^*+*^/*Hba-x*^*+*^),^39^ HSPCs (*CD34*^+^/*Cd27*^+^),^40,41^ myeloid/lymphoid/megakaryoid cells (*CD45*^+^, *ITGA2B*^+^/*Cd45*^+^, *Itga2b*^+^), Kupffer cells (*CD68*^+^, *C1QA*^+^/*Cd68*^+^, *C1qa*^+^),^42,43^ septum transversumal cells (STCs) (*PDGFRA*^*+*^, *NCAM1*^*+*^/*Pdgfra*^*+*^, *Ncam1*^*+*^),^3,6^ hepatic stellate cells (*DCN*^*+*^, *HGF*^+^/*Dcn*^*+*^, *Hgf*^+^),^3,6^ mesothelial cells (*PDPN*^*+*^/*Pdpn*^*+*^),^6^ and liver endothelial cells (*VEGFR3*^*+*^/*Vegfr3*^*+*^) (Fig. 1a, b). Erythropoiesis was extremely active in the fetal liver; hence, erythrocytes accounted for a large proportion of the total cells (Fig. 1a).

To understand the conservation and evolutionary differences in cell types between humans and mice, we performed a cross-species comparison analysis, which was based on one-to-one orthologues annotated by the Ensembl genome annotation system (http://www.ensembl.org/index.html). We found that 13 major cell types were relatively conserved according to the transcriptomic profiles in human and mouse fetal livers (Fig. 1c). Curiously, these cell types were clustered into four families, and based on the nature of the cell types involved, we designated these families as endoderm-derived lineages (hepatoblasts/hepatocytes and cholangiocytes), erythroid lineages (erythroid progenitors, erythroblasts, early erythrocytes, and primitive erythrocytes), non-erythroid hematopoietic lineages (HSPCs, Kupffer cells, and myeloid/lymphoid/megakaryoid cells), and mesoderm-derived non-hematopoietic lineages (hepatic stellate cells, STCs, mesothelial cells, and liver endothelial cells) (Fig. 1c).

Next, to study the conservation of and differences in gene regulatory networks between species, we used the WGCNA algorithm^44^ to construct weighted gene co-expression networks (WCGNs) based on genes differentially expressed in the 13 major cell types. To address differences caused by unbalanced sample sizes, we sampled 100 cells from each population to generate balanced datasets. Using these datasets, we constructed human and mouse topological overlap matrices (TOMs) to generate species-specific WCGNs. In both species, four cell family-specific gene modules clustered together (Supplementary information, Fig. S2a). We also calculated the centrality of each shared gene in human and mouse WCGNs and observed that the centralities were highly correlated between human and mouse (Supplementary information, Fig. S2b). These observations indicate that the regulatory hierarchy and positions of the regulators in the gene regulatory networks are conserved between humans and mice.

To study the conservation of and differences in gene expression patterns, we performed differential expression analysis on the resampled cells (Materials and Methods) to identify cell type-specific genes (Fig. 1d; Supplementary information, Table S2). Next, we focused on the abundant hepatoblasts/hepatocytes and erythroid progenitors to evaluate differences in gene expression patterns between species. We calculated the eigengenes of hepatoblast/hepatocyte- and erythroid progenitor-specific genes and found that the patterns of the eigengenes were conserved between humans and mice (Supplementary information, Fig. S2c). Despite these similarities, we also identified a series of genes that were specifically correlated with eigengenes of a single species, indicating the species-specific expression patterns of these genes, such as *ORM1*/*Orm1*, *NRN1*/*Nrn1* and *LDHB*/*Ldhb* (Supplementary information, Fig. S2d, e, Table S3).

### Identification of novel hepatic markers

**Fig. 2:**
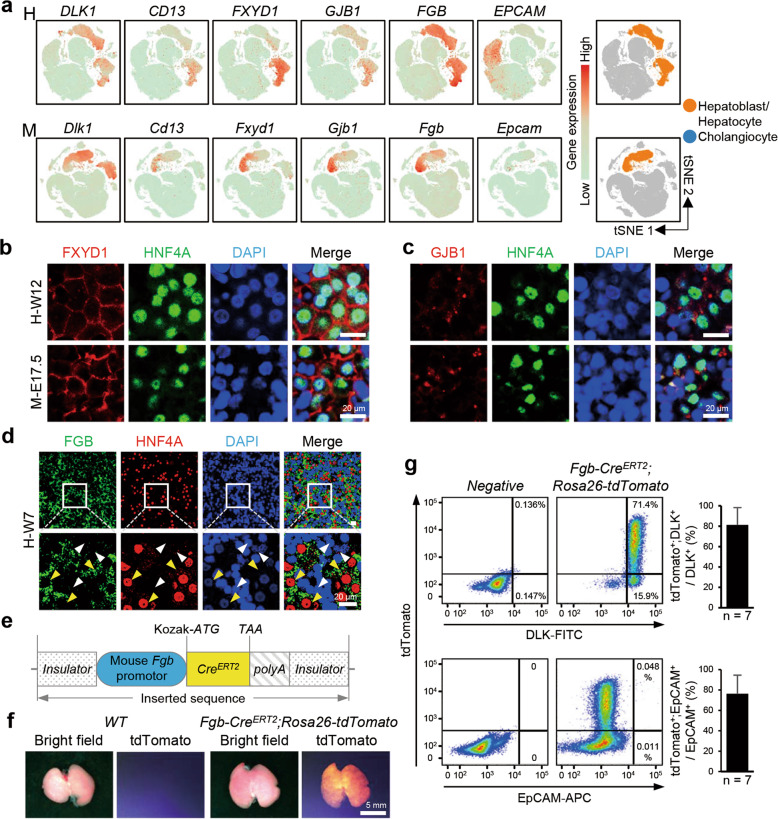
Identification of novel markers of hepatic cells. **a** t-SNE plots showing the expression levels of hepatobiliary marker genes. **b** Immunofluorescence showing the expression and distribution of FXYD1 and HNF4A in the W12 human (H-W12) and E17.5 mouse (M-E17.5) fetal livers. Scale bars, 20 μm. **c** Immunofluorescence showing the expression and distribution of GJB1 and HNF4A in the W12 human (H-W12) and E17.5 mouse (M-E17.5) fetal livers. Scale bars, 20 μm. **d** Immunofluorescence showing the expression and distribution of FGB and HNF4A in the W7 human (H-W7) fetal liver. The yellow arrowhead indicates the FGB^+^HNF4A^+^ hepatoblasts. The white arrowhead indicates the FGB^–^HNF4A^–^ cells. Scale bars, 20 μm. **e** Schematics of strategies for the generation of *Fgb-Cre*^*ERT2*^ transgenic mice. **f** Morphologies and tdTomato signals in the livers of E17.5 WT and *Fgb-Cre*^*ERT2*^*;Rosa26-tdTomato* mice. Scale bars, 5 mm. **g** FACS gating and statistical analysis showing the percentage of tdTomato^+^DLK^+^/DLK^+^ (upper) and tdTomato^+^EpCAM^+^/EpCAM^+^ (lower) cells in E17.5 WT and *Fgb-Cre*^*ERT2*^*;Rosa26-tdTomato* mice. n, number of embryos.

Next, we focused our analysis on endoderm-derived hepatic cells, including hepatoblasts, hepatocytes, and cholangiocytes (*SOX17*^+^ extrahepatic cholangiocytes were excluded from this study). Several cell surface markers, such as *CD13* and *DLK1*, were found to label and isolate hepatoblasts/hepatocytes from the mouse fetal liver.^45–47^ Based on our scRNA-seq data, we found that these marker genes were also expressed in the human fetal liver (Fig. 2a). Additionally, we identified new cell lineage-specific surface markers for hepatoblasts/hepatocytes, *FXYD1* and *GJB1*, in both the human and mouse fetal livers (Fig. 2a) and validated their expression in HNF4A^+^ hepatocytes in W12 human livers and E17.5 mouse livers by immunofluorescence (Fig. 2b, c). However, we found that *EPCAM*, a unique surface marker for cholangiocytes in mice, was not specific for cholangiocytes in the human fetal liver, as *EPCAM* was simultaneously expressed in both cholangiocytes and erythroid progenitors (Fig. 2a). In addition, the *FGB* gene encodes the fibrinogen β chain, and the gene expression profile database for E14.5 mouse embryos showed that *Fgb* was uniquely expressed in the liver (http://www.eurexpress.org). Single-cell transcriptomic analyses showed that *FGB* was specifically expressed in hepatobiliary cell lineages in both humans and mice (Fig. 2a). Immunostaining of FGB and HNF4A or the cholangiocyte marker SOX9 in W7 human embryonic liver sections revealed that FGB was co-distributed with HNF4A^+^ hepatoblasts/hepatocytes and the differentiated SOX9^+^ cells (Fig. 2d; Supplementary information, Fig. S3a). These findings suggested that a transgenic mouse strain expressing *Cre* recombinase under the control of the *Fgb* element can be used to trace and genetically manipulate hepatobiliary lineages. To test this hypothesis, we generated a transgenic mouse that contained the *Fgb* promoter adjoined to a sequence encoding inducible Cre recombinase (*Fgb-Cre*^*ERT2*^) (Fig. 2e). These mice were crossed with the *Rosa26-tdTomato* strain; pregnant *Fgb-Cre*^*ERT2*^;*Rosa26-tdTomato* mice were intraperitoneally injected with tamoxifen at E11.5, and embryos were investigated at E17.5. Compared with WT embryos, in *Fgb-Cre*^*ERT2*^*;Rosa26-tdTomato* embryos, tdTomato signals were exclusively detected in the liver but not in other major organs (Fig. 2f; Supplementary information, Fig. S3b). Flow cytometric analysis of liver cells showed that tdTomato marked an average of 81.9% of DLK^+^ hepatocytes and 76.9% of EpCAM^+^ cholangiocytes (Fig. 2g). To verify that *Fgb* is specifically expressed in tdTomato^+^ cells in the fetal livers of *Fgb-Cre*^*ERT2*^;*Rosa26-tdTomato* mice, we peritoneally injected tamoxifen at E11.5 and sorted tdTomato^+^ and tdTomato^*–*^ cells at E14.5 for single-cell reverse transcription quantitative PCR (RT-qPCR) to detect the expression levels of *Cre*^*ERT2*^, *Fgb*, *Alb*, and *Afp*. We observed that ~98% of tdTomato^+^ cells co-express *Cre*^*ERT2*^, *Fgb*, *Alb*, and *Afp*, but these genes could not be detected in tdTomato^–^ cells (Supplementary information, Fig. S3c, d). Fluorescence-activated cell sorting (FACS) plots also showed that ~94% of tdTomato^+^ cells expressed the hepatoblast marker DLK (Supplementary information, Fig. S3c). Taken together, these data indicate that Fgb is specifically expressed in hepatoblasts during the early stage of liver development and that *Fgb-Cre*^*ERT2*^ is an efficient tool for tracing and genetic manipulation of hepatoblasts. Therefore, these datasets provide a resource for identifying novel cell lineage-specific markers during hepatogenesis.

### Identification of a novel hepatoblast subpopulation

**Fig. 3:**
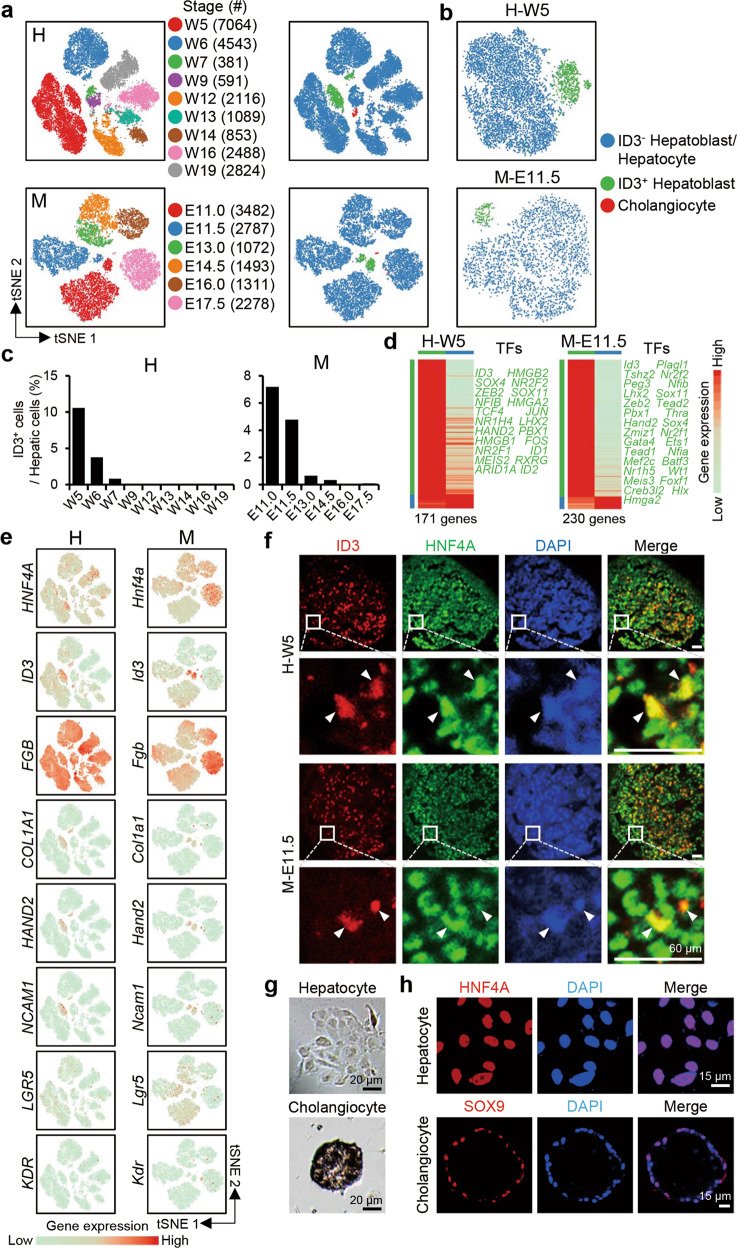
Identification of two hepatoblast subpopulations. **a** t-SNE plots showing the developmental stages (left) and clusters (right) of human (H) and mouse (M) endoderm-derived cells. **b** t-SNE plots showing the distinct clusters of ID3^+^ and ID3^–^ hepatoblasts in the W5 human (H-W5) and E11.5 mouse (M-E11.5) fetal livers. **c** The proportion of ID3^+^ cells in human (H) and mouse (M) hepatoblasts/hepatocytes at different developmental time points. **d** Differentially expressed genes in W5 human (H-W5) and E11.5 mouse (M-E11.5) ID3^+^ and ID3^–^ hepatoblasts. Each column represents a cell type and each row represents a gene. The TFs associated with each cell type are listed on the right. The color scheme is the same as **b**. **e** t-SNE plots showing the expression levels of marker genes. **f** Immunofluorescence showing the expression and distribution of ID3 and HNF4A in the W5 human (H-W5) and E11.5 mouse (M-E11.5) fetal livers. The arrowheads indicate ID3^+^ hepatoblasts. Scale bars, 60 μm. **g** The morphology of cultured hepatocytes (after 6-day culture from NCAM1^+^DLK^+^ hepatoblasts) and cholangiocyte tissue (after 10-day culture from NCAM1^+^DLK^+^ hepatoblasts). Scale bars, 20 μm. **h** Immunofluorescence showing the expression and distribution of HNF4A and SOX9 in cultured hepatocytes (upper) and cholangiocytes (lower), respectively. Scale bars, 15 μm.

On the t-distributed stochastic neighbor embedding (t-SNE) plot, the hepatoblasts/hepatocytes sampled at the same developmental time were generally clustered together, except the cells from the W5 and W6 human livers and the E11.0 and E11.5 mouse livers. At these early stages of liver development, from the same sample, we observed a small cell cluster separate from the main cluster (Fig. 3a, b). Our analyses revealed that 8% (918 cells of 11,604 cells) of W5–W6 human hepatoblasts and 6% (383 cells of 6269 cells) of E11.0–E11.5 mouse hepatoblasts belonged to this small cluster. However, after the early stages of liver development, this cluster could no longer be detected (Fig. 3c). Greater than 100 genes were differentially expressed between the small and the main clusters (Fig. 3d; Supplementary information, Table S4). Curiously, the small cluster of cells expressed many mesoderm-related genes, such as *ID3*, *COL1A1*, and *HAND2* (Fig. 3d, e), and gene ontology (GO) analysis showed that this cluster was enriched in genes involved in mesenchymal and epithelial development (Supplementary information, Fig. S4a and Table S4). To confirm the existence of this subgroup of hepatoblasts, we performed immunofluorescence to validate the existence of HNF4A^+^ID3^+^ cells in the W5 human fetal liver and E11.5 mouse fetal liver (Fig. 3f). We designated these HNF4A^+^ID3^+^ cells as ID3^+^ hepatoblasts. We also used a VEGFR3 antibody to mark the blood vessels but found that the distribution of HNF4A^+^ID3^+^ hepatoblasts was independent of that of blood vessels (Supplementary information, Fig. S4b). We reanalyzed our scRNA-seq data that was previously generated using the Smart-seq2 method^5^ and detected a few hepatoblasts with high expression of *Id3* (Supplementary information, Fig. S4c). However, due to the low cell number, these *Id3*^+^ cells could not be identified as an independent cell cluster.

To investigate the fate of ID3^+^ hepatoblasts, we collected ID3^+^ hepatoblasts to perform in vitro organoid culture. Our scRNA-seq data showed that *Ncam1* was highly expressed in ID3^+^ but not ID3^*–*^ hepatoblasts. FACS using an NCAM1 antibody could separate DLK^high^ mouse E11.5 hepatoblasts into two clusters, NCAM1^+^DLK^+^ and NCAM1^*–*^DLK^+^ (Supplementary information, Fig. S4d). NCAM1^+^DLK^+^ cells highly express *Id3* and were considered as ID3^+^ hepatoblasts (Supplementary information, Fig. S4e). Accordingly, NCAM1^*–*^DLK^+^ cells are ID3^*–*^ hepatoblasts. We also estimated the purity of our FACS-sorted cell population using single-cell RT-qPCR analysis and found it to be ~92.5% (Supplementary information, Fig. S4f). We then collected the same number of cells (5000) from the NCAM1^+^DLK^+^ and NCAM1^*–*^DLK^+^ populations and cultured them on gelatin.^48^ After 6 days, both NCAM1^+^DLK^+^ and NCAM1^*–*^DLK^+^ single hepatoblasts formed colonies and gave rise to HNF4A^+^ hepatocytes (Fig. 3g, h; Supplementary information, Fig. S4g). We also cultured NCAM1^+^DLK^+^ and NCAM1^*–*^DLK^+^ cells on Matrigel.^48^ After 10 days, a single hepatoblast formed a cystic structure comprised of SOX9^+^ cholangiocytes (Fig. 3g, h; Supplementary information, Fig. S4g). These data indicate that both ID3^+^ and ID3^*–*^ hepatoblasts contribute to liver development. Together, our data reveal a novel ID3^+^ hepatoblast subpopulation, although the origin of this subpopulation needs to be determined.

Recently, Prior et al.^14^ identified LGR5^+^ stem and progenitor cells from the hepatoblast pool at the early stage of liver development. We examined the expression patterns of *LGR5* and found a fraction of *LGR5*^+^ hepatoblasts based on our 10× analysis (Fig. 3e). Considering the dropping-out effect of the 10× platform, we also examined the expression pattern of *Lgr5* in the published Smart-seq2 datasets.^5^ Curiously, at E10.5–E13.5, 80%–90% of hepatoblasts express *Lgr5*, but the percentage of *Lgr5*^+^ cells dropped to ~40% or lower starting at E14.5 (Supplementary information, Fig. S5a–d). In cholangiocytes, *Lgr5* expression could not be detected (Supplementary information, Fig. S5a–d). This finding may suggest that *LGR5* expression is associated with bipotent developmental potential of hepatoblasts. However, on the tSNE plot of the 10× data or the PCA plot of the Smart-seq2 data, the *LGR5*^+^ and *LGR5*^–^ cells at a specific developmental stage are intermingled, indicating the transcriptomic similarity of these cells.

Suzuki et al.^49^ also identified CD29^+^CD49f^+/low^c-Kit^–^CD45^–^TER119^–^ stem cells in the fetal liver, which could be clonally propagated in vitro and generate hepatocytes and cholangiocytes. However, when we exanimated the expression patterns of these genes (*CD29*, *CD49F*, *KIT*, *CD45*, and TER119 coding gene *GYPA*) in our 10× and Smart-seq2 data, we found that hepatoblasts/hepatocytes possess these characteristic gene expression patterns (Supplementary information, Fig. S5e–g), indicating that the stem cells identified by Suzuki et al. were primarily hepatoblasts. Therefore, these two studies demonstrated the bipotentiality of hepatoblast function in vitro and in vivo, respectively.

### Conserved “default-directed” pathways of hepatoblast differentiation in humans and mice

**Fig. 4:**
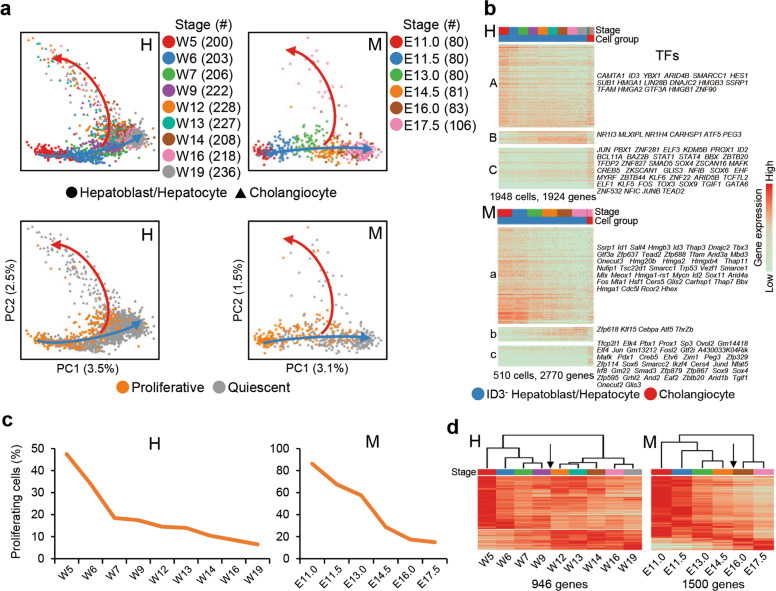
Conserved hepatoblast differentiation pathways between species. **a** Upper: PCA plots showing the differentiation pathways of human (H) and mouse (M) hepatobiliary cells. 250 and 80 hepatoblasts/hepatocytes were collected for human and mouse samples at each time point, respectively. Lower: PCA plots showing the proliferation of human (H) and mouse (M) hepatobiliary cells. Arrows indicate the directions of differentiation. **b** Heatmaps showing differentially expressed genes during human (H) and mouse (M) hepatoblast differentiation. Each column represents a cell and each row represents a gene. The TFs of each gene group are listed on the right. The color scheme is the same as **a**. **c** Changes in the proportion of proliferative cells (S and G2/M phases) in human (H) and mouse (M) hepatoblasts/hepatocytes. **d** Heatmaps showing differentially expressed genes and expression switches during human (H) and mouse (M) hepatoblast-to-hepatocyte development. Each column represents a stage and each row represents a gene. The color scheme is the same as **a**. Dendrograms showing the results of hierarchical clustering of different stages. Black arrows indicate the time point of cell fate transition.

Our previous scRNA-seq of marker-enriched hepatobiliary cells illustrated that hepatoblast differentiation in mice follows a “default-directed” model in which the hepatoblast-to-hepatocyte transition is a progressive process characterized by gradual changes in gene expression, but the hepatoblast-to-cholangiocyte transition is a specifically regulated process.^5^ However, whether human hepatoblasts adopt similar strategies to differentiate is unclear. We performed differentiation trajectory analysis of hepatobiliary cells using human and mouse 10× Genomics datasets. As hepatoblasts/hepatocytes comprised the majority of detected endoderm-derived cells in our unbiased dataset, we performed sampling to obtain a small number of hepatoblasts/hepatocytes and ensured that cholangiocyte-related genes could be uncovered by a variable gene-finding algorithm. We then performed principal component analysis (PCA) of the sampled hepatoblasts/hepatocytes with all detected cholangiocytes. In the PCA plots of both human and mouse cells, the hepatoblast-to-hepatocyte transition formed a main straight trajectory along PC1, while cholangiocytes followed dispersed branches along PC2 (Fig. 4a). This pattern was similar to that identified in our previous experiments in mice using the Smart-seq2 method. Additionally, ID3^+^ hepatoblasts were located near or overlapping with ID3^–^ hepatoblasts and maintained the same developmental progress in pseudotime along PC1 (Supplementary information, Fig. S5h). After excluding cell cycle-related genes, heatmaps from differential expression analysis showed that group-A/a and group-B/b genes were gradually downregulated or upregulated during hepatoblast-to-hepatocyte differentiation, whereas cholangiocytes specifically expressed group-C/c genes, including many TFs, such as *SOX4*, *SOX9*, and *SOX6*, in both humans and mice (Fig. 4b; Supplementary information, Table S5). These results suggested that in both species, hepatoblasts default to the hepatocyte fate and follow a gradual and progressive transition to hepatocytes, while cholangiocyte differentiation requires escape from this default fate choice via de novo activation of regulatory factors.

Using the CellCycleScoring algorithm,^50^ we identified the cell cycle phases of human and mouse hepatoblasts/hepatocytes (Supplementary information, Fig. S5i). Consistent with a previous study in mice, the proportion of proliferative hepatoblasts/hepatocytes (the cells in S and G2/M phases) decreased through human hepatoblast/hepatocyte development. Specifically, these proliferative cells decreased from W5 (~50%) to W7 (< 20%) in humans, and at W19, the proportion of proliferative cells was less than 10% (Fig. 4a, c). Therefore, in both humans and mice, the proliferation rate of hepatoblasts/hepatocytes decreased throughout development.

In our previous study, we found that the hepatoblast-to-hepatocyte transition was completed after E14.5. Hierarchical clustering analysis of 10× Genomics data also detected a dramatic switch in gene expression between E14.5 and E16.0 (Fig. 4d), which is consistent with our previous study.^5^ Similarly, hierarchical clustering analysis strongly suggested that the hepatoblast-to-hepatocyte transition was completed at a time point between W9 and W12 (Fig. 4d). Altogether, these analyses revealed conserved pathways and characteristics between humans and mice during hepatic lineage differentiation.

### Heterogeneity of human fetal hepatocytes

**Fig. 5:**
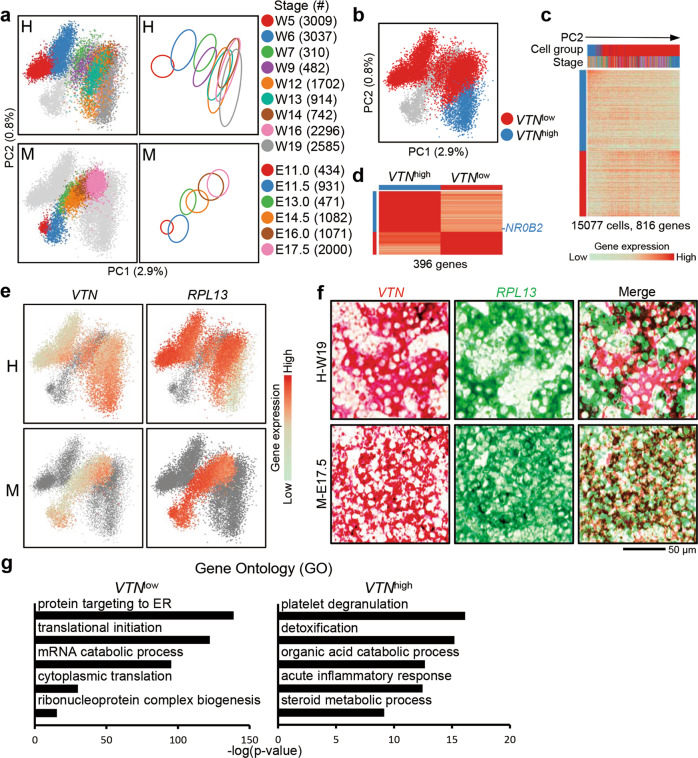
Heterogeneity of quiescent human hepatocytes. **a** Left: PCA plots of human (H) and mouse (M) quiescent hepatoblasts/hepatocytes. Right: Schematic summary of quiescent hepatoblast/hepatocyte development based on the PCA plots. **b** PCA plots showing the clustering of human *VTN*^low^ and *VTN*^high^ hepatocytes based on PC2-related genes. **c** Heatmap showing the expression of PC2-related genes in human hepatoblasts/hepatocytes ordered by PC2 values. Each column represents a cell and each row represents a gene. The color scheme of cell stages is the same as **a**. **d** Differentially expressed genes in *VTN*^low^ and *VTN*^high^ hepatocytes. The TFs of each gene group are listed on the right. **e** Expression levels of the marker genes *VTN* and *RPL13* in human (H) and mouse (M) hepatoblasts/hepatocytes. **f** In situ hybridization of sequential sections showing the distribution of *VTN* and *RPL13* in the W19 human (H-W19) and E17.5 mouse (M-E17.5) fetal livers. Scale bars, 50 μm. **g** GO analysis of genes differentially expressed in W19 human *VTN*^low^ and *VTN*^high^ hepatocytes.

Based on scaled orthologous genes, we performed PCA of quiescent hepatoblasts/hepatocytes from humans and mice (Materials and Methods). All cells were arranged according to their developmental process along PC1 (Fig. 5a). At the earlier stages of liver development in both humans and mice, ID3^–^ hepatoblasts/hepatocytes displayed a nearly homogenous distribution. However, in the fetal livers of humans but not mice, the distribution of developing hepatocytes gradually expanded along PC2 (Fig. 5a). We classified two cell groups based on the PC2-related gene expression patterns (Fig. 5b, c; Supplementary information, Table S6). Differential expression analysis showed that one cell group highly expresses the vitronectin coding gene *VTN*, which is mainly expressed in the liver rather than other organs (https://www.proteinatlas.org/ENSG00000109072-VTN/tissue) (Fig. 5d; Supplementary information, Table S6). We named this group of cells *VTN*^high^ cells, and correspondingly, the other group was named *VTN*^low^ cells. To validate the heterogeneity of the maturing human hepatocytes, we analyzed the expression patterns of *RPL13*, a gene highly expressed in *VTN*^low^ cells, and *VTN* in the fetal liver by in situ hybridization. In the W19 human liver, *VTN*^*+*^ and *RPL13*^*+*^ hepatocytes formed separate cell clusters that generally presented a mutually exclusive spatial distribution, unrelated to the positions of the portal veins (PVs) or central veins (CVs). In contrast, *Vtn*^*+*^ and *Rpl13*^*+*^ cells were widely distributed and colocalized in E17.5 mouse hepatocytes (Fig. 5e, f).

Differential gene expression and GO analyses of *VTN*^high^ and *VTN*^low^ hepatocytes indicated that highly expressed genes in *VTN*^high^ cells were enriched for liver functions, including blood coagulation, detoxification, and organic acid metabolism, while genes highly expressed in *VTN*^low^ cells were associated with growth and increases in cell mass and had functions in processes such as ribosome synthesis and protein translation (Fig. 5g; Supplementary information, Table S6). Notably, at each biological developmental stage, all of these maturing hepatocytes maintained the same developmental pace along maturation pseudotime (PC1) (Fig. 5a). In mice, maturing hepatocytes from each biological stage clustered tightly and displayed homogeneous expression of *Vtn* and *Rpl13* (Fig. 5a, e, f).

In the adult liver, hepatocytes are heterogeneously distributed along the axis of the CV and PV. This unique distribution is called zonation, which organizes metabolism and secretion in a highly hierarchical structure and facilitates efficient fulfillment of liver functions. To investigate whether this fetal hepatocyte heterogeneity reflects zonation-related hepatocyte heterogeneity, we identified CV and PV cells based on the expression patterns of zonation-related genes described in previous scRNA-seq studies of the adult human liver^2^ and projected the adult CV and PV cells to the PCA plot of human fetal hepatocytes. Adult CV and PV hepatocytes were intermingled along the PC2, and zonation-related genes, such as *GLUL* and *HAL*, did not show distinct expression patterns in *VTN*^low^ and *VTN*^high^ cells (Supplementary information, Fig. S6a, b). This result indicates that fetal gene expression heterogeneity is not related to zonation.

In humans, a rare *EPCAM*^+^*NCAM1*^+^ cell population in W15–W19 and adult livers was recently reported to be putative liver stem cells named hepatobiliary hybrid progenitors (HHyPs), which have the potential to differentiate into hepatocytes and cholangiocytes.^13,51^ We projected the scRNA-seq data of HHyPs on the PCA plot of hepatobiliary development, and surprisingly, we found that HHyPs were projected onto the cholangiocyte population (Supplementary information, Fig. S7a, b). This result suggested that HHyPs are cholangiocytes or retain the characteristics of cholangiocytes.

### Erythroid lineage differentiation and maturation pathway in the fetal liver

**Fig. 6:**
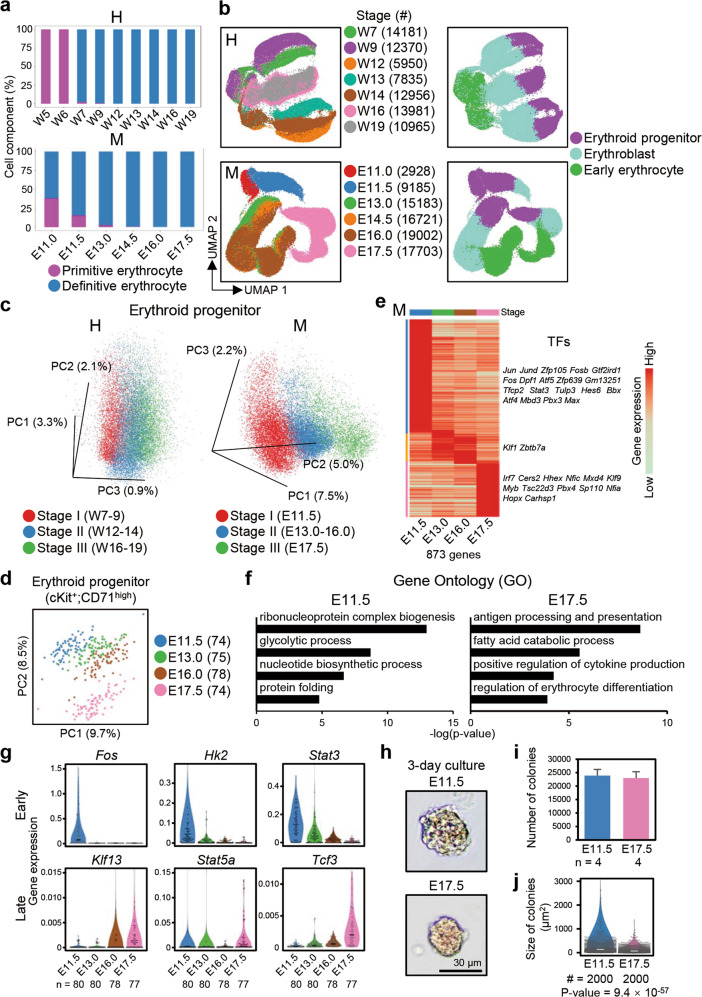
Erythropoiesis in the fetal liver. **a** The proportion of primitive erythrocytes in human (H) and mouse (M) whole erythrocytes at different developmental time points. **b** UMAP plots showing developmental stages and cell types during erythropoiesis in the human (H) and mouse (M) fetal livers. **c** 3D-PCA plots showing the maturation of human (H) and mouse (M) erythroid progenitors. **d** PCA plots of mSTRT-seq data showing the maturation of E11.5–E17.5 mouse cKit^+^CD71^high^ cells. **e** Heatmap of mSTRT-seq data showing differentially expressed genes in E11.5–E17.5 mouse erythroid progenitors. Each column represents a stage and each row represents a gene. The TFs of each stage are listed on the right. **f** GO analysis of genes differentially expressed in cKit^+^CD71^high^ E11.5 and E17.5 cells. **g** Violin plots showing the expression levels of marker genes of E11.5–E17.5 mouse cKit^+^CD71^high^ cells validated by single-cell RT-qPCR. The y-axis represents the relative expression values normalized to *Gapdh* expression. Each dot represents a single cell. The black line within each violin plot indicates the median of the expression levels. n, number of single cells. **h** The morphology of E11.5 and E17.5 mouse cKit^+^CD71^high^ cells cultured for 3 days. Scale bars, 30 μm. **i** Statistical analysis showing the number of colonies for cultured E11.5 and E17.5 mouse cKit^+^CD71^high^ cells. n, number of biological replicates. **j** Statistical analysis showing the size of the colonies for cultured E11.5 and E17.5 mouse cKit^+^CD71^high^ cells. The white line indicates the median size of colonies. #, number of randomly selected colonies used to determine the size statistics.

The vast majority of cells in the fetal liver were hematopoietic cells, which were mainly comprised of erythroid cells. *HBE1*^+^/*Hba-x*^+^ yolk sac-derived erythrocytes existed prior to W9 in humans and E14.5 in mice as the primitive erythrocyte population rapidly decreased (Fig. 6a). Then, AGM-derived definitive erythrocyte population rapidly increased. In this study, we focused on the process of definitive erythropoiesis. We presented cell types at different stages of erythropoiesis on the UMAP (Fig. 6b). At a given developmental time, we observed an erythropoiesis path along erythroid progenitor–erythroblast–early erythrocytes. Interestingly, erythroid progenitors existed at all detected time points but displayed different transcriptional profiles, especially in the human fetal liver, suggesting that a transition in progenitor features occurs throughout fetal development. However, after differentiation into early erythrocytes, the gene expression patterns generally converged (Fig. 6b). In both humans and mice, erythropoiesis could be divided into three different stages: W7–W9, W12–W14, and W16–W19 in humans and E11.5, E13.0–E16.0, and E17.5 in mice. Erythroid cells from the same stage clustered together (Fig. 6b).

Focusing on the erythroid progenitors at different stages, we performed three-dimensional (3D) PCA and identified developmental pathways of erythroid progenitor development in both humans and mice; these pathways may reflect the developmental process of fetal erythroid progenitors (Fig. 6c). To confirm the transcriptomic differences between erythroid progenitors, we used FACS to isolate colony-forming units-erythroids (CFU-Es) (cKit^+^CD71^high^)^52^ from E11.5, E13.0, E16.0, and E17.5 mouse livers and performed scRNA-seq using a more sensitive well-based modified STRT-seq (mSTRT-seq) approach^53^ (Supplementary information, Fig. S8a). This mSTRT-seq produced single-cell cDNA and involved barcoding during reverse transcription. Then, the samples were pooled for library construction to avoid batch effects caused during library construction and sequencing. In total, we obtained 301 cells after quality control, with an average of 200,000 unique molecular identifiers (UMIs) and greater than 4000 genes (Supplementary information, Fig. S8b). The sorted CFU-Es expressed erythroid progenitor markers *Kit*, *Cd71*, and *Gata1* (Supplementary information, Fig. S8c). Similarly, we observed the separation of cell populations at E11.5, E13.0, E16.0, and E17.5 in the PCA plot (Fig. 6d), and differential expression analysis identified stage-specific genes (Fig. 6e; Supplementary information, Table S7). GO analysis revealed that genes highly expressed at E11.5 were mainly associated with cell growth and proliferation, which included processes such as glycolysis and biogenesis of ribonucleoprotein and nucleotides, while genes highly expressed at E17.5 were involved in erythrocyte differentiation and terminal functions (Fig. 6f). Moreover, the *Aldoa*, *Fos*, *Hk2*, and *Stat3* genes, which are important in regulating progenitor characteristic and proliferation,^54–57^ were highly expressed at E11.5, whereas the genes *Klf13*, *Stat5a*, and *Tcf3*, which play roles in regulating erythroid cell differentiation,^58–60^ were highly expressed at E17.5. The expression patterns of these genes and other genes (*Aldoa* and *Galk1*, *Fech* and *Gsta4*, and *Cela1* and *Cirbp*, which were highly expressed in the early, middle, and late developmental stages, respectively) were validated by single-cell RT-qPCR (Fig. 6g; Supplementary information, Fig. S8d).

CFU-Es have the ability to proliferate and can directly generate proerythroblasts, the first erythroid cells recognizable by morphology.^61^ For functional validation, we collected CFU-Es for colony-forming unit (CFU) assays. We isolated the same number (100,000) of CFU-Es from E11.5 and E17.5 mouse livers and cultured the single cells in MethoCult GF M3434 medium. After 3 days, we found that there was no difference in the numbers of colonies between the cultured erythroid progenitors from different stages. However, the size of colonies from E11.5 erythroid progenitors was significantly larger than that from E17.5 erythroid progenitors, indicating that the early erythroid progenitors retain a high proliferation rate (Fig. 6f, h–j). Altogether, these results demonstrate that erythroid progenitors are not stable in their own state and that they are developing while differentiating into erythrocytes.

### Non-erythroid hematopoietic lineage differentiation pathway

**Fig. 7:**
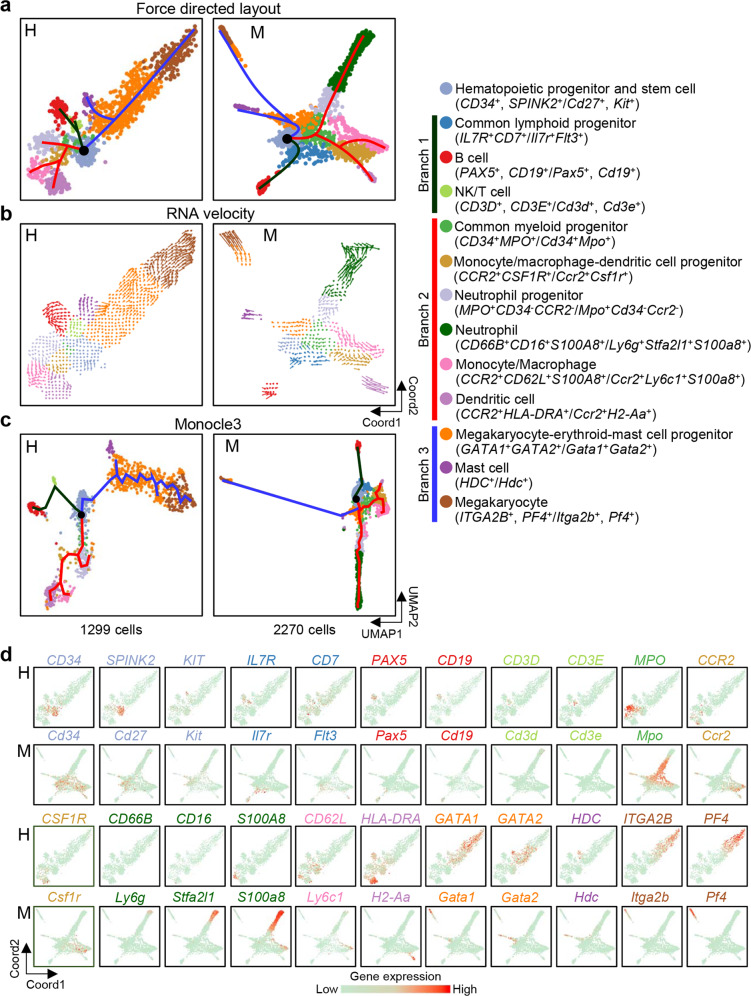
Hematopoietic pathways in the fetal liver. **a**–**c** FDL (**a**), RNA velocities (**b**) and Monocle3 UMAP plots (**c**) showing the developmental trajectories of hematopoiesis in the human (H) and mouse (M) fetal livers. Lines indicate the directions of differentiation. **d** FDL plots showing the expression levels of hematopoietic lineage marker genes.

We next focused on non-erythroid hematopoietic cells. Using Seurat,^50^ we identified 13 cell types in our second round of cell type classification: HSPCs (*CD34*^+^, *SPINK2*^+^/*Cd27*^+^, *Kit*^+^),^40,41,62^ common lymphoid progenitors (*IL7R*^+^*CD7*^+^/*Il7r*^+^*Flt3*^+^),^63–65^ B cells (*PAX5*^+^, *CD19*^+^/*Pax5*^+^, *Cd19*^+^),^66^ NK/T cells (*CD3D*^+^, *CD3E*^+^; not identified in mice),^67^ common myeloid progenitors (*CD34*^+^*MPO*^+^/*Cd34*^+^*Mpo*^+^),^68,69^ monocyte/macrophage-dendritic cell progenitors (*CCR2*^+^*CSF1R*^+^/*Ccr2*^+^*Csf1r*^+^),^70–72^ neutrophil progenitors (*MPO*^*+*^*CD34*^*–*^*CCR2*^*–*^*/Mpo*^*+*^*Cd34*^*–*^*Ccr2*^*–*^),^70,73^ neutrophils (not identified in humans; *Ly6g*^+^*Stfa2l1*^+^*S100a8*^+^ in mice),^74–77^ monocytes/macrophages (*CCR2*^*+*^*CD62L*^*+*^*S100A8*^*+*^*/Ccr2*^*+*^*Ly6c1*^*+*^*S100a8*^*+*^),^31,70,77,78^ dendritic cells (*CCR2*^+^*HLA-DRA*^+^/*Ccr2*^+^*H2-Aa*^+^),^31,70,71^ megakaryocyte-erythroid-mast cell progenitors (*GATA1*^+^*GATA2*^+^/*Gata1*^+^*Gata2*^+^),^79^ mast cells (*HDC*^+^/*Hdc*^+^),^80^ and megakaryocytes (*ITGA2B*^+^, *PF4*^+^/*Itga2b*^+^, *Pf4*^+^)^31,81,82^ (Fig. 7a–d). Notably, contrary to the stage-specific patterns of erythroid progenitor development, at W7–W19 in humans and E13.0–E16.0 in mice, the cells were generally clustered together (Supplementary information, Fig. S9a, b), indicating that non-erythroid hematopoietic cells were continuously produced and did not show developmental stage-related changes in gene expression. Using force-directed layout, we analyzed the cells at W7–W19 in humans and E13.0–E16.0 in mice and delineated three HSPC-derived lineage differentiation branching paths (Fig. 7a). We also applied the RNA velocity algorithm^83^ and Monocle3^84^ to predict the developmental directions of the cell lineages (Fig. 7b, c). The HSPC-derived lineage differentiation branching paths, which were consistent with those described in a previous scRNA-seq study in humans, were conserved in humans and mice.^31^ Although neutrophils were not identified in the human fetal liver, we found that a large percentage of blood cells in the mouse liver are neutrophils. In addition, we identified a *CD3E*^+^ NK/T cell population in human but not mouse fetal livers (Fig. 7a–d). These analyses identified differences in lineage components between human and mouse hematopoiesis. Overall, single-cell analysis of human and mouse non-erythroid hematopoietic cell lineages enabled us to determine the similarities and differences between species.

### Generation of Kupffer cells, endothelial cells, and mesenchymal lineages in the liver

Next, we focused on mesoderm-derived liver-resident cell types, including Kupffer cells and non-hematopoietic lineages of liver endothelial cells and liver mesenchymal lineages (hepatic stellate cells, mesothelial cells, and STCs).^23^ Together, these cells accounted for only a small percentage of cells in the fetal liver (~2.7% in the W19 human fetal liver or ~3.4% in the E17.5 mouse fetal liver). The t-SNE plot showed that the distributions of these mesoderm-derived liver-resident cell types at different developmental time points did not overlap, indicating that the maturation process occurred across fetal development (Supplementary information, Fig. S9a, b).

Based on the expression patterns of the tissue-specific endothelial marker genes *LYVE1*/*Lyve1* (sinusoidal endothelial cells), *ITGA2*/*Itga*2 (PVs and arteries), *ITGA3*/*Itga3* (PVs), and *ITGB4*/*Itgb4* (CVs and arteries), we found that vascular endothelial cells (*ITGA2*^+^/*Itga*2^+^ or *ITGA3*^+^/*Itga3*^+^ or *ITGB4*^+^/*Itgb4*^*+*^) accounted for a small proportion of the endothelial cells we identified, while sinusoidal endothelial cells (*LYVE1*^+^/*Lyve1*^*+*^) accounted for most of the endothelial cells (Supplementary information, Fig. S9c). This finding may be due to the fact that the digestive conditions used in this study are not suitable for isolation of vascular endothelial cells.

**Fig. 8:**
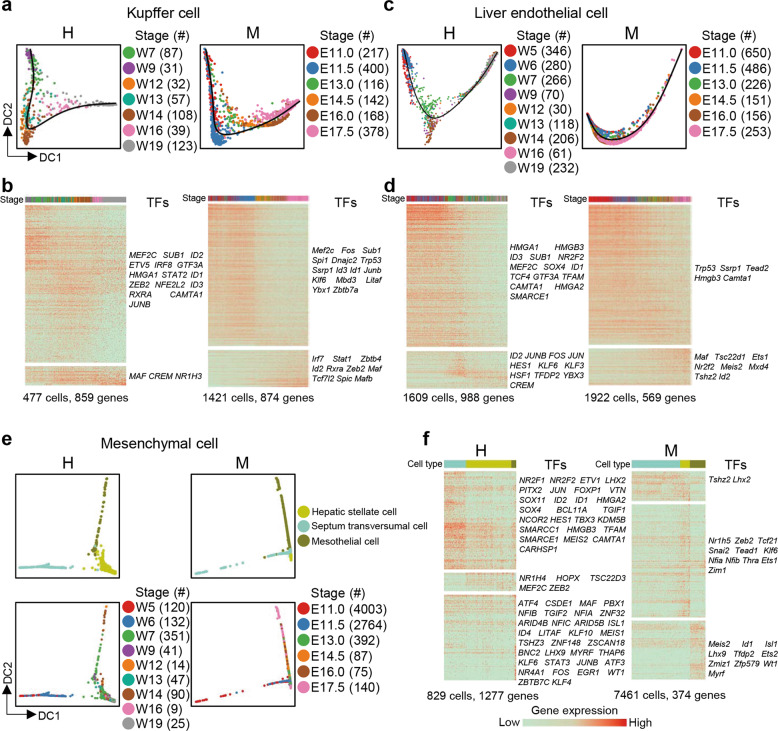
Developmental pathways of mesoderm-derived cells. **a** Diffusion maps showing the developmental pathways of Kupffer cells in the human (H) and mouse (M) fetal livers. **b** Heatmaps showing the differentially expressed genes of Kupffer cells in the human (H) and mouse (M) fetal livers. Each column represents a cell and each row represents a gene. The color scheme is the same as **a**. The TFs of each gene group are listed on the right. **c** Diffusion maps showing the developmental pathways of liver endothelial cells in the human (H) and mouse (M) fetal livers. **d** Heatmaps showing the differentially expressed genes of liver endothelial cells in the human (H) and mouse (M) fetal livers. The color scheme is the same as **c**. **e** Diffusion maps showing the developmental pathways of hepatic stellate cells, septum transversumal cells, and mesothelial cells in the human (H) and mouse (M) fetal livers. **f** Heatmaps showing the differentially expressed genes during mesenchymal cell differentiation in the human (H) and mouse (M) fetal livers. The color scheme is the same as **e**.

To investigate the developmental process of Kupffer cells, liver endothelial cells, and liver mesenchymal cells, we performed diffusion mapping on these cell clusters. We defined the continued maturation pathways of Kupffer cells and liver endothelial cells in humans and mice, and using hierarchical clustering analysis, we identified genes that were differentially expressed during the human and mouse maturation processes (Fig. 8a–d; Supplementary information, Table S8). Liver mesenchymal cells were comprised of hepatic stellate cells, STCs, and mesothelial cells, all of which exhibited clearly distinct differentiation paths on the diffusion map. At early developmental stages, STCs differentiated into hepatic stellate cells and mesothelial cells, which then entered maturation processes (Fig. 8e). We identified cell type-specific genes expressed during liver mesenchymal cell differentiation (Fig. 8f; Supplementary information, Table S8). Therefore, using high-throughput scRNA-seq, we detected mesoderm-derived cell populations with fewer cells in the fetal liver and identified their differentiation and maturation pathways.

## Discussion

Fetal liver development is a complex process that includes differentiation, immigration, and interaction of many cell lineages derived from the endoderm and mesoderm. Our understanding of the overall landscape of fetal liver development is limited. Using scRNA-seq, we described the comprehensive cell composition and developmental pathways of the human and mouse fetal livers, mainly focusing on the processes that comprise hepatogenesis. The major cell types and developmental pathways were generally similar between humans and mice; however, we observed significant differences between species in terms of the gene expression patterns, heterogeneity, and composition of certain cell types.

In the W5–W6 human and E11.0–E11.5 mouse fetal livers, we identified a new subpopulation of ID3^+^ hepatoblasts that expressed many mesenchymal-featured genes (Fig. 3a, b, d, e). These ID3^+^ hepatoblasts consisted of a considerable proportion of hepatoblasts at early stages of development that then rapidly decreased to undetectable levels. In the early stages of liver bud formation, the septum transversum mesenchyme surrounds and invades the nascent liver bud. These ID3^+^ hepatoblasts may be differentiated from STCs or affected by STCs or other signals to express mesenchyme-related genes. The origin of ID3^+^ hepatoblasts requires further investigation.

In our previous study, we used antibodies against DLK and EpCAM to sort mouse hepatic lineages for low-throughput smart-seq2 scRNA-seq,^5^ and here, we performed high-throughput analysis using the 10× Genomics platform. The main conclusions that mouse hepatoblast differentiation follows the “default-directed” model and the time point of hepatoblast-to-hepatocyte transition were consistent between these studies. This study confirmed that the “default-directed” model of hepatoblast differentiation that was developed in mice is also applicable to human. However, after W12, human hepatocytes exhibited heterogeneity; some hepatocytes displayed stronger protein-producing capacity (*VTN*^low^), and others were more functionally specialized (*VTN*^high^) (Fig. 5a–g). Notably, this heterogeneity is unrelated to hepatocyte zonation (Supplementary information, Fig. S6a, b). This heterogeneity is absent in murine fetal hepatocytes. In fact, mouse hepatocytes are more similar to the human *VTN*^low^ state (Fig. 5a) and do not display heterogeneity (Fig. 5a, e, f). These differences indicated that, in contrast to mouse hepatocytes, a portion of human hepatocytes may become functionally active at the early stages of fetal liver development.

Several studies have suggested the existence of liver stem cells in the fetal liver. Bipotential liver stem cells may differentiate into both hepatocytes and cholangiocytes or contribute to transdifferentiation between the two cell types.^49,51,85^ Combining the recent scRNA-seq studies^13^ and the marker genes of the identified fetal liver progenitors, we found that these progenitors are likely hepatoblasts or a fraction of cholangiocytes that may retain potential to differentiate into hepatocytes. Prior et al.^14^ identified LGR5^+^ stem and progenitor cells from the hepatoblast pool at the early stage of liver development, and Suzuki et al.^49^ also identified stem cells (CD29^+^CD49f^+/low^c-Kit^–^CD45^–^TER119^–^) in fetal livers. Given these findings and those from our scRNA-seq analysis, we concluded that these cells represent hepatoblasts. Recently, Segal et al.^13^ performed scRNA-seq of sorted NCAM1^+^ HHyPs. When we projected their scRNA-seq data to our data, we found that those NCAM1^+^ cells were located within the cholangiocyte population (Supplementary information, Fig. S7a). Curiously, a previous lineage tracing study showed that a subset of fetal Sox9^+^ cholangiocytes can revert to hepatocyte fate during development,^85^ and a study identified human hepatic stem cells located in the ductal plates in the fetal liver. This finding suggests that cholangiocytes at the early developmental stage maintain plasticity to transdifferentiate into hepatocytes. In this study, we uncovered a subpopulation of ID3^+^ hepatoblasts only at the earliest stage of hepatogenesis. ID3^+^ and ID3^–^ cells are separate cell clusters on the tSNE plot, indicating that the ID3^+^ cell population is distinct from the ID3^–^ population. We also validated that both ID3^+^ and ID3^*–*^ hepatoblasts could give rise to hepatocytes and cholangiocytes in an in vitro culture system. Based on these findings, we conclude that we identified a novel bipotent hepatoblast population.

Few studies have focused on the development of mesoderm-derived non-hematopoietic lineages in the human fetal liver. In our study, we found that the differentiation or maturation pathways of resident STCs, liver endothelial cells, and Kupffer cells followed a timeline in both the human and mouse fetal livers, similar to the hepatoblast-to-hepatocyte differentiation pathway (Figs. 4a, b and 8). In addition, we discovered that erythroid progenitors experience stage changes at the transcriptomic level (Fig. 6b–f). In contrast, many other hematopoietic lineages (non-erythroid) in the fetal liver follow a developmental stage-independent process during W7–W19 in humans or E13.0–E16.0 in mice in which the non-erythroid hematopoietic cell lineages maintain relatively stable expression profiles (Supplementary information, Fig. S9a). Nevertheless, we observed that the proportions of various blood lineages differed in humans and mice (Fig. 7a–d).

Together, our findings provide a clear and comprehensive understanding of the differentiation processes of all cell types in the human and mouse fetal livers. Defining the developmental pathways of various cell types in vivo provides key data needed to direct cell lineage differentiation and liver organoid construction in vitro.

## Materials and methods

### Mouse lines

All experimental animal protocols were approved by the Institutional Animal Care and Use Committee of Peking University. All mice were maintained under pathogen-free conditions at 23 ± 2 °C with a 12-h day/night cycle. C3H male and C57BL/6 female mice were purchased from Vital River Laboratories (Beijing, China). F1 progenies of C3H and C57BL/6 mice were used. The morning on which the vaginal plug was detected was defined as E0.5. *Rosa26-tdTomato* mice^86^ were used for tracing.

The *Fgb-Cre*^*ERT2*^ transgenic mouse strain was generated in this study. Briefly, the mouse *Fgb* promoter (~11.0 kb from the translation start codon) adjoined to the coding sequence of inducible Cre recombinase *Cre*^*ERT2*^ was cloned into the pInsulator vector. The target region of the *pInsulator-Fgb-Cre*^*ERT2*^ fragment on the final vector was released by I-CeuI restriction enzyme digestion and injected into the pronucleus of mouse zygotes. Zygotes were transplanted into surrogate mice. Transgenic integration was examined by PCR. Genetically stable transgenic mice were obtained after at least five generations of mating. Eight- to twelve-week-old *Rosa26-tdTomato* females were mated with *Fgb-Cre*^*ERT2*^ males and intraperitoneally injected with tamoxifen (Cayman Chemical, #13258, 4 mg/20 g body weight) at E11.5.

### Human embryos

Human embryos were obtained following electively terminated pregnancies at Haidian Maternal & Child Health Hospital in Beijing. All experiments were performed in accordance with protocols approved by the Peking University Institutional Review Board (PU-IRB) (certificate number: IRB00001052-18083). Written informed consent was obtained before sample collection. The gender of human tissues was determined based on the expression patterns of the Y-chromosome gene *RPS4Y1*.

### Cell sample preparation

For 10× Genomics scRNA-seq, human or mouse fetal livers were dissociated into single cells by treatment with 0.25% trypsin at 37 °C. The reaction was terminated by the addition of 0.4 volumes of FBS. Cells were washed once with PBS and passed through a 50-μm filter into a new tube. For mSTRT-seq, mouse fetal livers were directly pipetted to release blood cells. Cells were sequentially passed through 70-μm and 50-μm filters into new tubes.

### scRNA-seq library preparation

For droplet-based scRNA-seq, cDNA preparation and library construction were conducted using the Single Cell 3′ Reagent Kit v2 (10× Genomics) according to the manufacturer’s instructions. For well-based mSTRT-seq, a single targeted cell was picked up for barcoded cell lysis, and cDNAs from 48–96 different cells were pooled together. Four cycles of PCR amplification using biotinylated index primers were conducted to produce 3′ end biotin-tagged cDNA. After cDNA fragmentation using a Bioruptor plus (Diagenode), biotin-tagged fragments were captured using “Dynabeads MyOne Streptavidin C1” beads (Thermo, 65002), and libraries were constructed using a Kapa Hyper Prep Kit (Kapa Biosystems, KK8505). The qualities of cDNAs and libraries were assessed using an Advanced Analytical Fragment Analyzer (AATI).

### Immunofluorescence staining

Dissected human or mouse liver tissues were fixed in 4% paraformaldehyde at 4 °C for at least 12 h and incubated in 30% sucrose at 4 °C overnight. Samples were embedded in the optimum cutting temperature (OCT) compound and cut into 5-μm sections. After blocking with TBST containing 20% FBS, sections were incubated with anti-FXYD1 (Abcam, ab76597, 1:500), anti-GJB1 (Abcam, ab66613, 1:500), anti-HNF4A (Santa Cruz Biotechnology, sc-6556, 1:50), anti-SOX9 (Millipore, ab5535, 1:200), anti-FGB (Abcam, ab118533, 1:500), anti-ID3 (Abcam, ab41834, 1:500), and anti-VEGFR3 (Invitrogen, 14-5988-82, 1:100) antibodies at 4 °C overnight. After washing, sections were treated with Alexa Fluor 488 donkey anti-goat IgG (Invitrogen, A11055, 1:1000), Alexa Fluor 594 donkey anti-goat IgG (Invitrogen, A11058, 1:1000), Alexa Fluor 488 donkey anti-rabbit IgG (Invitrogen, A11008, 1:1000), Alexa Fluor 594 donkey anti-rabbit IgG (Invitrogen, A21207, 1:1000), Alexa Fluor 647 donkey anti-rat IgG (Jackson ImmunoResearch, 712-605-150, 1:250), and Alexa Fluor 488 donkey anti-sheep IgG (Invitrogen, A11015, 1:1000). DAPI (Sigma, D9564, 0.5 µg/mL) was used for nuclear staining. Images were acquired using an LSM 710 NLO and DuoScan System (Zeiss).

### In situ hybridization

The cDNA templates were derived from human or mouse fetal liver tissues. Total RNA was extracted using the RNeasy Micro Kit (Qiagen, 74004) and was reverse transcribed with SuperScript II Reverse Transcriptase (Invitrogen, 18064014). T7 and SP6 promoter sequences were attached to the 5′ end of gene-specific primers used to create probes. Probe templates were synthesized using X5 Plus High-Fidelity DNA Polymerase PCR Mix (2×) (Mei5 Biotechnology, MF006), cloned into the pTOPO-Blunt vector (Mei5 Biotechnology, MF021), validated by sequencing, and linearized with *Eco*RI (New England Biolabs, R3101). Riboprobes were generated using the DIG RNA Labeling Kit (Roche, 11277073910).

Dissected W19 human and E17.5 mouse liver tissues were fixed in 4% paraformaldehyde at 4 °C for at least 12 h and then incubated in 30% sucrose and 4% paraformaldehyde at 4 °C overnight. Fixed samples were embedded in OCT reagent and cut into 5-μm sequential sections. In situ hybridization was performed according to a previously described protocol with some modifications.^87^ Sections were refixed in 4% paraformaldehyde at room temperature for 20 min. After washing twice with PBS, sections were incubated in 70% ethanol at 4 °C for at least 1 h. After washing, sections were treated with proteinase K (Invitrogen, 25530049, 1:1000) in PBST; the proteinase K reaction was terminated with 2 mg/mL glycine. Antisense riboprobes were used for hybridization, and BM purple was used for signal detection. Images were acquired on an AXIOIMAGER M2 fluorescence microscope (Zeiss).

### Flow cytometry

Human or mouse liver single-cell suspensions were incubated with anti-DLK-FITC (MBL, D187-4, 1:100), anti-EpCAM-APC (eBioscience, 17-5791-82, 1:50), anti-NCAM1 (Sangon Biotech, D198946, 1:50), Alexa Fluor 647 donkey anti-mouse IgG (Invitrogen, A31571, 1:1000), anti-cKit-APC (BioLegend, 105812, 1:200), or anti-CD71-PE (eBioscience, 113808, 1:200) antibodies at 4 °C for 20 min. After washing once with PBS, cells were analyzed and sorted using a FACS Aria SORP cell sorter (BD Biosciences). Dead cells were excluded by DAPI staining.

### RT-qPCR

For single-cell RT-qPCR, cDNA from individual cells was diluted with water in a ratio of 1:20. RT-qPCR was performed using 2× M5 HiPer Realtime PCR Super mix with Low Rox (Mei5 Biotechnology, MF797) on a Roche LightCycler^®^ 480 Instrument II. For bulk-cell RT-qPCR, total RNA was extracted from the collected cells using the RNAprep Pure Micro kit (Tiangen, DP420), and reverse transcription was performed with HiScript II Q RT SuperMix for RT-qPCR (Vazyme, R223-1). Primer sequences are listed in Supplementary information, Table S9.

### Hepatoblast differentiation in vitro

For hepatocyte differentiation, the collected hepatoblasts were plated on gelatin-coated (Sigma) 24-well dishes for 6 days. For cholangiocyte differentiation, the collected hepatoblasts were plated on Matrigel-coated (BD Bioscience) 24-well dishes for 10 days. Cells were cultured in DMEM/F12 (Gibco) medium supplemented with 10% FBS, 1× ITS-X (Invitrogen), 1× Penicillin-Streptomycin (Gibco), 25 ng/mL hEGF, 25 ng/mL hHGF, and 40 ng/mL dexamethasone (Sigma). For immunostaining, cells were stained with anti-SOX9 (Millipore, ab5535, 1:200), anti-HNF4A (Santa Cruz Biotechnology, sc-6556, 1:50), Alexa Fluor 488 donkey anti-goat IgG (Invitrogen, A11055, 1:1000), and Alexa Fluor 488 donkey anti-rabbit IgG (Invitrogen, A11008, 1:1000). DAPI (Sigma, D9564, 0.5 µg/mL) was used for nuclear staining. Images were acquired using an LSM 710 NLO and DuoScan System (Zeiss).

### Mouse CFU assays

Collected cells were washed twice with Iscove’s MDM plus 2% FBS, added to MethoCult GF M3434 medium (Stemcell Technologies), and vortexed. Cells were plated on 12-well plates and incubated for 3 days. Images were acquired on an AXIOIMAGER M2 fluorescence microscope (Zeiss).

### scRNA-seq and data preprocessing

The 10× Genomics libraries were sequenced as 150-bp paired-end reads on the Illumina HiSeq 4000 platform. Raw files were processed with Cell Ranger 2.0.2 using the default parameters. Human and mouse reads were respectively mapped to the GRCh38 or mm10 reference genomes version 1.2.0 provided by 10× Genomics (https://support.10xgenomics.com/single-cell-gene-expression/software/downloads/latest). Cell filtering was performed with Cell Ranger using the default settings.

### Preprocessed data normalization and variable gene identification

Preprocessed data were imported into the Seurat v2.2.1 R package.^50^ The UMI counts of each cell were normalized using the “NormalizeData” function with a scale factor of 10,000. Then, variable genes were identified using the “FindVariableGenes” function.

### Dimension reduction, RNA velocity, and trajectory analysis

PCA was performed based on the variable genes. We further performed t-SNE based on the PCA subspaces.

For the developmental trajectory visualization, we applied three independent algorithms: FDL, UMAP, and diffusion map. Before dimensional reduction, we performed hierarchical clustering of variable genes and removed cell cycle-related genes and transcripts from contaminated blood cells. FDL was performed using the R package igraph v1.2.2 (https://cran.r-project.org/web/packages/igraph/) based on the shared nearest neighbor (SNN) matrix, which was built using the “BuildSNN” function in Seurat. UMAP and diffusion map analyses were performed using the functions “RunUMAP” and “RunDiffusion” in Seurat, respectively. We fitted the development pathway with a principal curve (smoothing spline fitness) using princurve v2.1.3 (https://cran.r-project.org/web/packages/princurve/).

RNA velocity was determined using velocyto v0.17.16^83^ with default parameters and analyzed using the function “gene.relative.velocity.estimates” in the R package velocyto.R v0.6^83^ with parameters “kCells = 10, fit.quantile = 0.02”. The embedding of arrows representing RNA velocity was performed using the function “show.velocity.on.embedding.cor” in velocyto.R with parameters “*n* = 30, scale = sqrt’”.

Trajectory analysis was performed using the R package Monocle3 v0.2.0.^84^ The preprocessing and dimension reduction were performed using the functions “preprocess_cds” and “reduce_dimension” in Monocle3 with default parameters. The trajectories were calculated using the function “learn_graph” in Monocle3 with the parameter “minimal_branch_len = 2” based on defined cell clusters.

### Cell clustering, doublet identification, batch effect correction, and differential expression analysis

Cell clustering was performed with the whole dataset using the “FindClusters” function in Seurat. Three major cell populations of endoderm-derived cells, erythrocytes, and other mesoderm-derived cells were separately isolated as subset data. Each population was subjected to next round of clustering using the same procedure. To identify detailed cell subtypes in non-erythroid blood cells, we separately isolated E11.0–E11.5, E13.0–E16.0, and E17.5 mouse non-erythroid blood cells and W7–W19 human non-erythroid blood cells from other mesoderm-derived cells and performed clustering.

Doublet identification was performed using the following method. First, to simulate the transcriptomic state of doublets, we randomly sampled 10% of cells from the whole dataset twice to form two cell subsets. Cells in two subsets were merged to make artificial doublets. We performed PCA on the union of the artificial doublets and the whole datasets using the Seurat standard pipeline. Based on the PCA space, the proportion of artificial doublets in the top 1% of cells nearest to each cell was considered the cell’s doublet index. Cells with doublet indices in the top 10% were considered putative doublets.^88^ Next, we performed differential expression analysis between putative doublets and normal cells to define doublet-related genes. Dimension reduction and clustering were performed based on the doublet-related genes using the Seurat standard pipeline. The clusters with high putative doublet percentages were defined as definitive doublets. For other mesoderm-derived non-erythroid cells, we identified hemoglobin genes enriched in the PC using PCA and performed clustering based on hemoglobin-related genes. Then, doublets derived from erythrocytes were identified and removed.

To eliminate batch effects among different stages, we performed batch effect correction using the “scanorama.correct” function in the Python package Scanorama^89^ with default parameters.

To overcome biases due to unbalanced sample sizes, we searched the differentially expressed genes of 13 cell populations using the following procedure. We sampled 100 cells per cell population to form a balanced dataset. To minimize disturbances due to dropping out, we performed imputation of this dataset using scImpute^90^ with default parameters. Then, differential expression analysis was performed on this dataset using the function “FindAllMarkers” in Seurat. To ensure robustness, we repeated the sampling and differential expression analysis 50 times. Differentially expressed genes with *P* values < 10^–20^ were recorded. Differentially expressed genes that were reproducible with 50 replicates were defined as specific differentially expressed genes in the 13 cell populations.

To determine the differentially expressed genes on the development pathways, we performed differential expression analysis among various developmental stages, and classified the differentially expressed genes using hierarchical clustering. Next, we used a heatmap to present gene clusters related to development processes (cell cycle-related genes and contaminated blood cell-related genes were filtered out).

### Cross-species comparison and weighted correlation network analysis

Our cross-species comparison was based on one-to-one orthologues annotated by the Ensembl genome annotation system (http://www.ensembl.org/index.html). The average expression levels in 13 major cell types were calculated. Pearson correlations of 13 major cell types in human and mouse were used to determine cross-species similarities.

Weighted correlation network analysis was performed using the WGCNA algorithm.^44^ To overcome biases due to unbalanced sample sizes, we sampled 100 cells per cell population to form a balanced dataset. Based on the differentially expressed genes in the 13 major cell types, we constructed human and mouse TOMs to generate species-specific WCGNs.

To determine the similarity and difference between human and mouse WCGNs, we calculated the centrality of each shared gene in the human and mouse WCGNs, which was equal to the sum of the neighbor edge weights of a node in the WCGN. Pearson correlation between the human and mouse centralities was used to evaluate the structure similarity between human and mouse WCGNs. To identify the different expression patterns of hepatic cells and erythroid cells, we individually calculated the eigengene of each cell type’s differentially expressed genes.^44^ Next, for each gene, we calculated the gene-to-human eigengene and gene-to-mouse eigengene Pearson correlations. Genes specifically correlated with the eigengene of one species (Pearson correlations > 0.5) but not correlated with the other (the differences between Pearson correlations of two species > 0.5) were regarded as having species-specific expression patterns.

### Identification of proliferative cells in hepatoblasts/hepatocytes

We identified the cell cycle phases of hepatoblasts/hepatocytes using the “CellCycleScoring” function in Seurat with the parameters “g2m.genes = g2m.genes, s.genes = s.genes, n.bin = 10”. The reference genes used to determine the S and G2/M phases were based on the default cell cycle-related gene list in Seurat.

### Analysis of hepatoblast differentiation pathways

Due to the unbalanced population sizes of hepatocytes and cholangiocytes, we first performed sampling of hepatoblasts/hepatocytes. According to the size of the cholangiocyte population, we collected 250 hepatoblasts/hepatocytes at each stage for human samples and 80 hepatoblasts/hepatocytes at each stage for mouse samples. The sampled hepatoblasts/hepatocytes were concatenated with all cholangiocytes and underwent PCA dimensional reduction. We calculated the Pearson correlation between PC1/2 scales and gene expression values to define PC1/2-related genes. Next, hierarchical clustering was performed on PC1/2-related genes to identify hepatocyte- and cholangiocyte-related genes.

### Combination of human and mouse hepatoblasts/hepatocytes

To analyze the heterogeneity of human and mouse hepatoblasts/hepatocytes, we combined the human and mouse datasets using the following procedure. First, we excluded species-specific genes of the human and mouse datasets based on orthologous genes annotated by the Ensembl genome annotation system (http://www.ensembl.org/index.html). Next, we separately identified the variable genes and performed scaling on gene expression matrices from the human and mouse datasets to reduce the species-derived difference. The scaled matrices from humans and mice were combined, and PCA was performed based on the union set of human and mouse variable genes.

### Analysis of mSTRT-seq data

The mSTRT-seq libraries were sequenced as 150-bp paired-end reads on an Illumina HiSeq 4000 platform. Raw files (fastq format) were separated based on cell-specific barcode sequences. PolyA sequences were trimmed from R1 reads (3′ end of cDNA). Preprocessed R1 reads from each cell were then aligned to the *Mus musculus* reference genome GRCm38/mm10 with TopHat V2.1.0. Bam files were annotated to genes with featureCounts (v1.5.3).^91^ We used samtools (v1.3.1)^92^ to sort and index the output bam files. UMIs of each gene were counted with umi-tools (v0.5.0)^93^ with the “unique” method. Next, the UMI count matrix was imported into Seurat v2.2.1 and transformed into transcripts per 0.1 million (TP0.1M), which normalized the number of total transcripts in one single-cell library to 100,000. Then, TP0.1M was further ln-normalized [lnTP0.1M]. Cells with < 4000 detected genes were removed from further analyses. Dimension reduction and differential expression analysis were performed using Seurat as described previously.

### GO enrichment analysis

GO enrichment analysis was performed using the R package clusterProfiler v3.10.0.^94^

### Statistics

A two-tailed Wilcoxon rank-sum test was used to compare differentially expressed genes between populations and analyze single-cell RT-qPCR results.

## Supplementary information

Supplementary information, Figure S1

Supplementary information, Figure S2

Supplementary information, Figure S3

Supplementary information, Figure S4

Supplementary information, Figure S5

Supplementary information, Figure S6

Supplementary information, Figure S7

Supplementary information, Figure S8

Supplementary information, Figure S9

Supplementary information, Table S1

Supplementary information, Table S2

Supplementary information, Table S3

Supplementary information, Table S4

Supplementary information, Table S5

Supplementary information, Table S6

Supplementary information, Table S7

Supplementary information, Table S8

Supplementary information, Table S9

## Data Availability

The RNA-seq data from this publication have been deposited in the Genome Sequence Archive (GSA) and assigned the identifiers CRA002443 and CRA002445.
