# Supplementary material for: Comparative analysis of cell lineage differentiation during hepatogenesis in humans and mice at the single-cell transcriptome level
Source: Cell Res. 2020 Jul 20;30(12):1109–26. doi: 10.1038/s41422-020-0378-6 (PMC7784864; doi:10.1038/s41422-020-0378-6)
Supplement: Supplementary file 1 — Supplementary information, Figure S1 [file 41422_2020_378_MOESM1_ESM.pdf]

Wang/Xu Figure S1

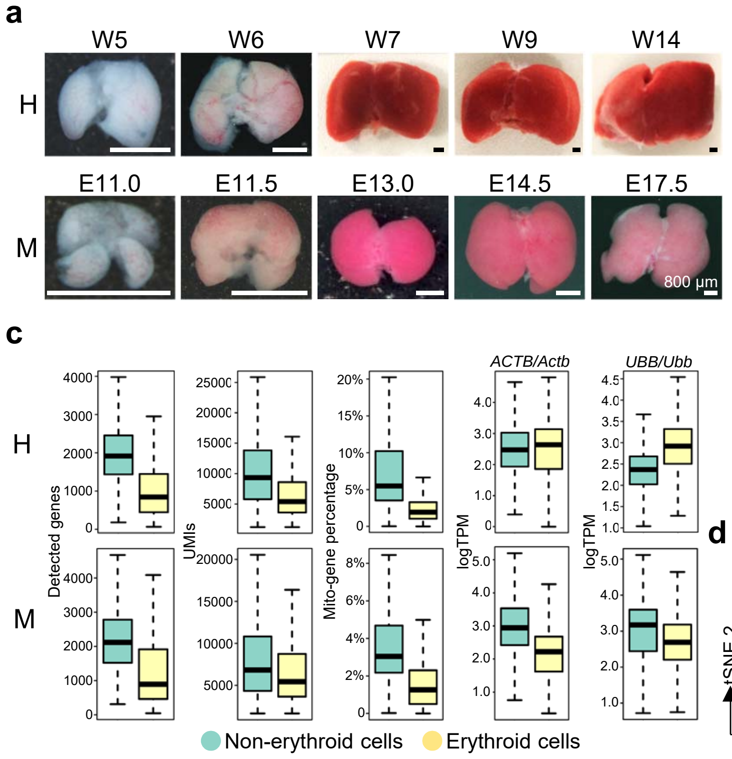

b

Species	Stage	Gender	Cell #
Human	W5	Male	8,706
	W6	Female	6,516
	W7	Female	16,541
	W9	Male	13,747
	W12	Female	8,948
	W13	Male	10,048
	W14	Male	15,500
	W16	Male	18,414
	W19	Male	17,542
	Total		115,962
Mouse	E11.0	Mix	14,708
	E11.5	Mix	20,499
	E13.0	Mix	19,112
	E14.5	Mix	21,211
	E16.0	Mix	23,121
	E17.5	Mix	28,182
	Total		126,833

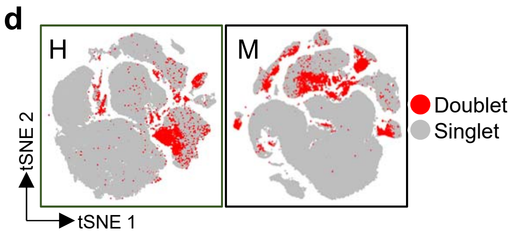

Supplementary information, Fig. S1 Quality control for scRNA-seq of human and mouse fetal livers. a Images of human (H) and mouse (M) fetal livers. Scale bars, 800 µm. **b** The sample list, including species, stage, gender, and captured cell number. **c** Boxplots showing detected genes, UMIs, mitochondrial-gene percentage and the expression of housekeeping genes *ACTB*/*Actb* and *UBB*/*Ubb* in human (upper) and mouse (lower) non-erythroid cells and erythrocytes. **d** t-SNE plots showing doublet distribution in 10× Genomics data from human (H) and mouse (M) fetal livers.
