# Supplementary material for: Comparative analysis of cell lineage differentiation during hepatogenesis in humans and mice at the single-cell transcriptome level
Source: Cell Res. 2020 Jul 20;30(12):1109–26. doi: 10.1038/s41422-020-0378-6 (PMC7784864; doi:10.1038/s41422-020-0378-6)
Supplement: Supplementary file 2 — Supplementary information, Figure S2 [file 41422_2020_378_MOESM2_ESM.pdf]

Wang/Xu Figure S2

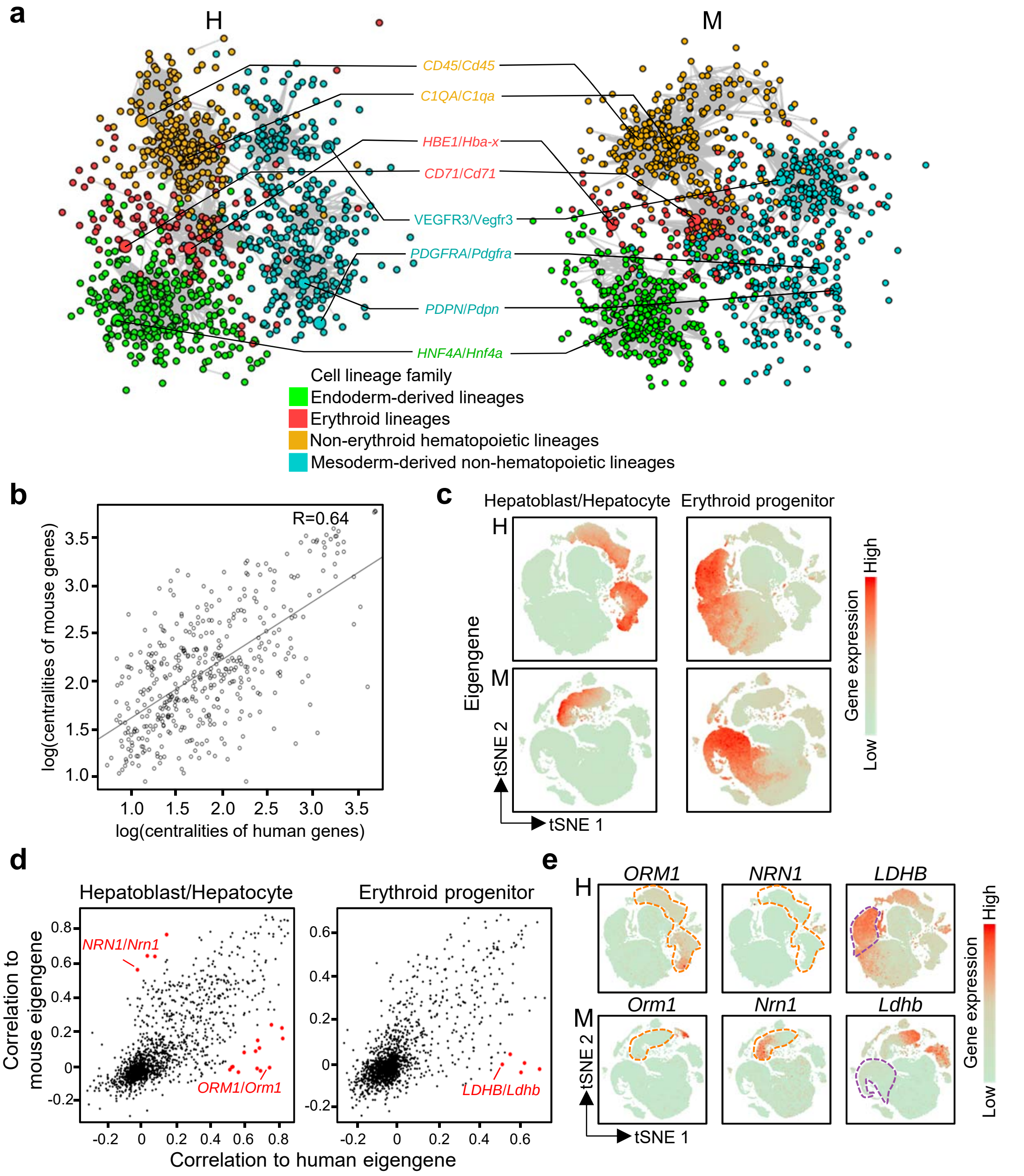

Supplementary information, Fig. S2 Cross-species analysis of weighted gene co-expression networks and gene expression patterns. a Weighted gene co-expression networks (WGCNs) of human (H) and mouse (M) cells. Colored dots indicate differentially expressed genes among the four cell lineage families within the species. Polylines point out the marker genes among the four cell lineage families. **b** Correlation between the centralities of human and mouse WGCNs. **c** Eigengenes of hepatoblasts/hepatocytes and erythroid progenitors in human (H) and mouse (M) samples. **d** Correlations of genes with human or mouse eigengenes associated with hepatoblasts/hepatocytes or erythroid progenitors. Red dots indicate genes for which the correlation to one species was 0.5 higher than the other species and the correlation itself was higher than 0.5. **e** t-SNE plots showing the expression levels of three genes identified in **d**.
