# Supplementary material for: Comparative analysis of cell lineage differentiation during hepatogenesis in humans and mice at the single-cell transcriptome level
Source: Cell Res. 2020 Jul 20;30(12):1109–26. doi: 10.1038/s41422-020-0378-6 (PMC7784864; doi:10.1038/s41422-020-0378-6)
Supplement: Supplementary file 3 — Supplementary information, Figure S3 [file 41422_2020_378_MOESM3_ESM.pdf]

Wang/Xu Figure S3

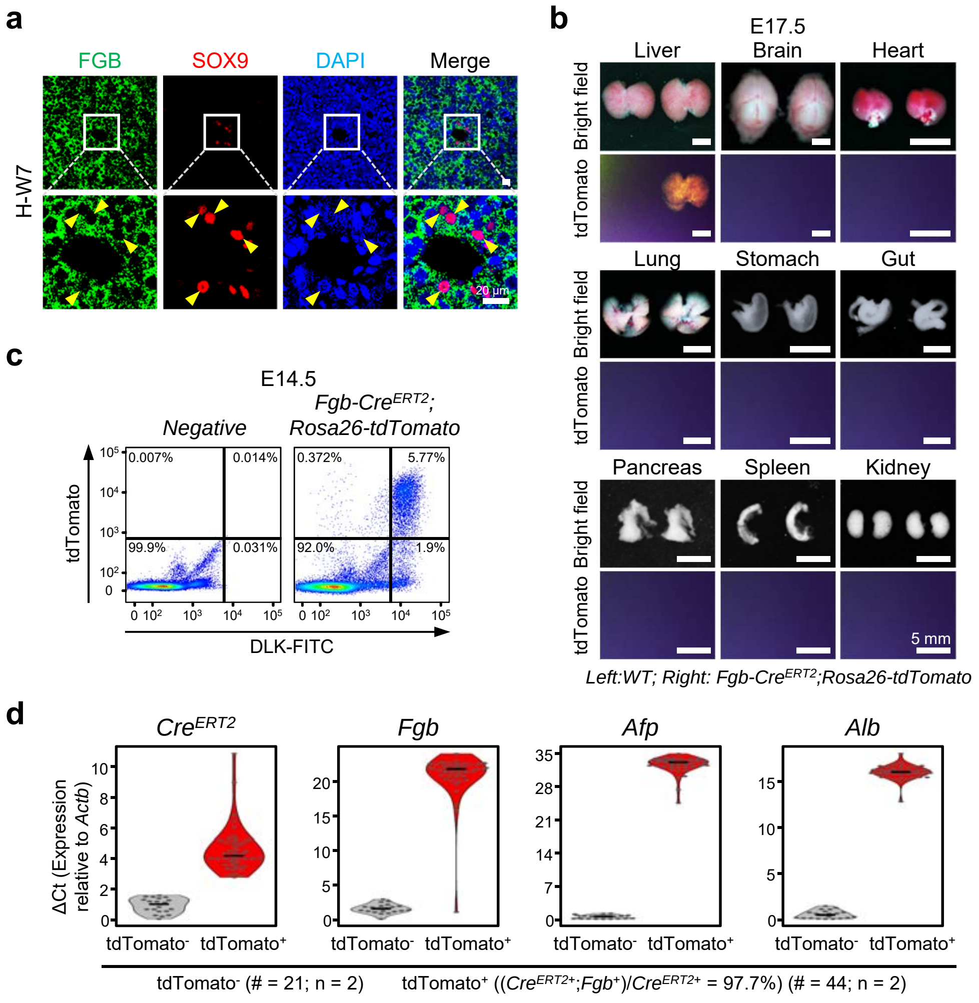

Supplementary information, Fig. S3 Examination of labeling efficiency of *Fgb-Cre*$^{\mathbf{ERT2}}$ transgenic mice. a Immunofluorescence showing the expression and distribution of FGB and SOX9 in the W7 human (H-W7) fetal liver. The yellow arrowhead indicates $FGB^{+}SOX9^{+}$ cholangiocytes. Scale bars, 20 μm. **b** Morphologies and tdTomato signals in several major organs of E17.5 *WT* and *Fgb-Cre^{ERT2};Rosa26-tdTomato* mice. Scale bars, 5 mm. **c** FACS gating of $tdTomato^{-}$ and $tdTomato^{+}$ cells in E14.5 *WT* and *Fgb-Cre^{ERT2};Rosa26-tdTomato* mice. **d** Violin plots showing the expression levels of *Cre^{ERT2}*, *Fgb*, *Alb*, and *Afp* in $tdTomato^{-}$ and $tdTomato^{+}$ cells from E14.5 *Fgb-Cre^{ERT2};Rosa26-tdTomato* mice quantified by single-cell RT-qPCR. Each dot represents a single cell. #, number of single cells. n, number of embryos.
