# Supplementary material for: Comparative analysis of cell lineage differentiation during hepatogenesis in humans and mice at the single-cell transcriptome level
Source: Cell Res. 2020 Jul 20;30(12):1109–26. doi: 10.1038/s41422-020-0378-6 (PMC7784864; doi:10.1038/s41422-020-0378-6)
Supplement: Supplementary file 4 — Supplementary information, Figure S4 [file 41422_2020_378_MOESM4_ESM.pdf]

Wang/Xu Figure S4

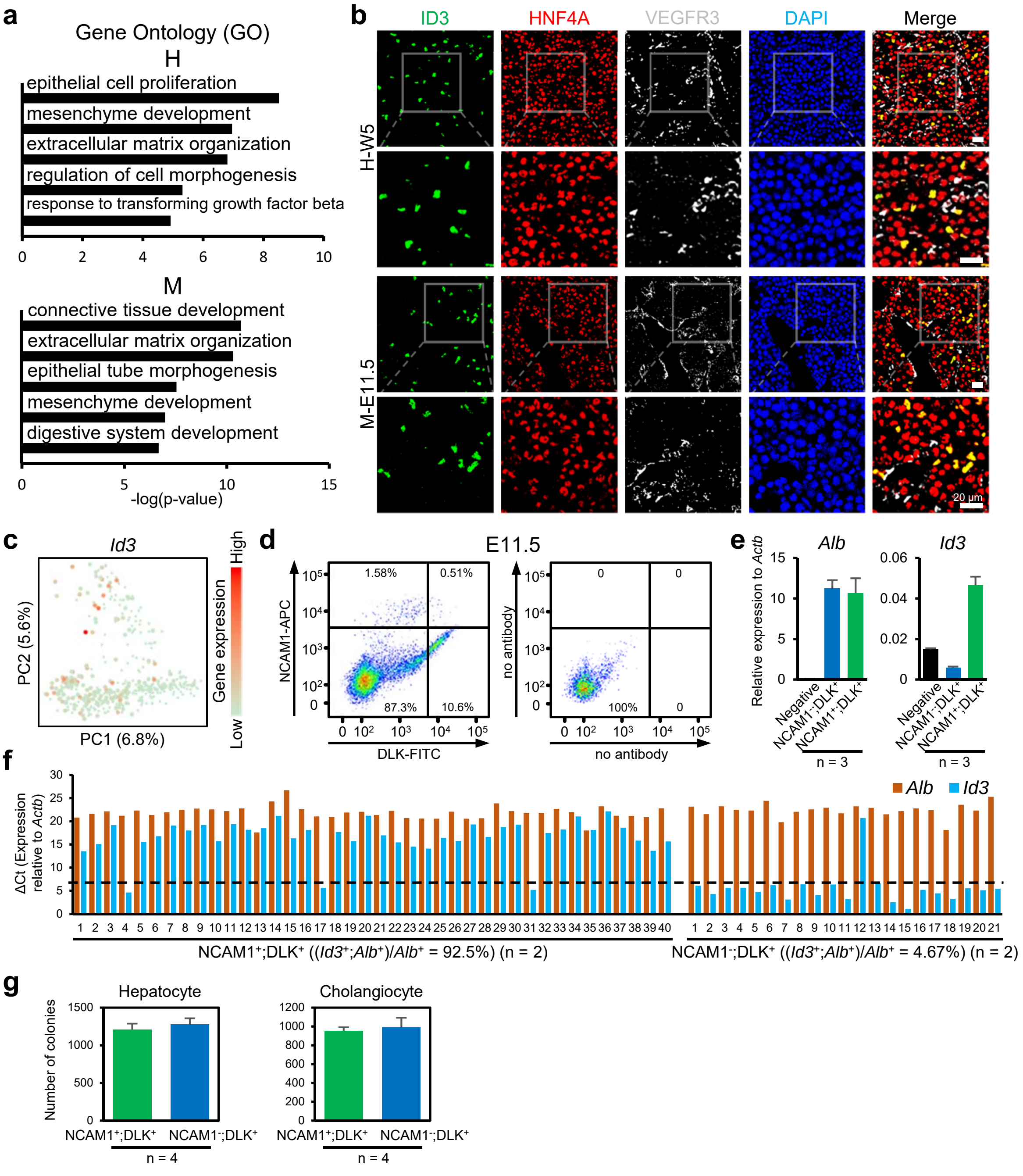

Supplementary information, Fig. S4 Identification and functional verification of ID3$^+$ hepatoblasts. a GO analysis of genes highly expressed in human (H) and mouse (M) ID3$^+$ hepatoblasts. **b** Immunofluorescence showing the expression and distribution of ID3 and HNF4A in the W5 human (H-W5) and E11.5 mouse (M-E11.5) fetal liver. VEGFR3 marks vessels. Scale bars, 20 μm. **c** PCA plot showing *Id3* expression in a previous mouse hepatoblast differentiation Smart-seq2 dataset.[5] **d** FACS gating showing the expression and distribution of NCAM1 and DLK on E11.5 mouse liver cells. **e** RT-qPCR analysis showing the expression of marker genes *Alb* and *Id3* in cells from each cluster of E11.5 mouse liver. n: number of embryos. **f** Single-cell RT-qPCR analysis estimating the percentage of *Id3*$^+$ hepatoblasts in NCAM1$^+$DLK$^+$ and NCAM1$^-$DLK$^+$ cells. The dashed line indicates the threshold of *Id3* expression. n: number of embryos. **g** Statistical analysis showing the number of colonies of the cultured hepatocytes (6-day culture) and cholangiocytes (10-day culture). n, number of biological replicates.
