# Supplementary material for: Comparative analysis of cell lineage differentiation during hepatogenesis in humans and mice at the single-cell transcriptome level
Source: Cell Res. 2020 Jul 20;30(12):1109–26. doi: 10.1038/s41422-020-0378-6 (PMC7784864; doi:10.1038/s41422-020-0378-6)
Supplement: Supplementary file 5 — Supplementary information, Figure S5 [file 41422_2020_378_MOESM5_ESM.pdf]

Wang/Xu Figure S5

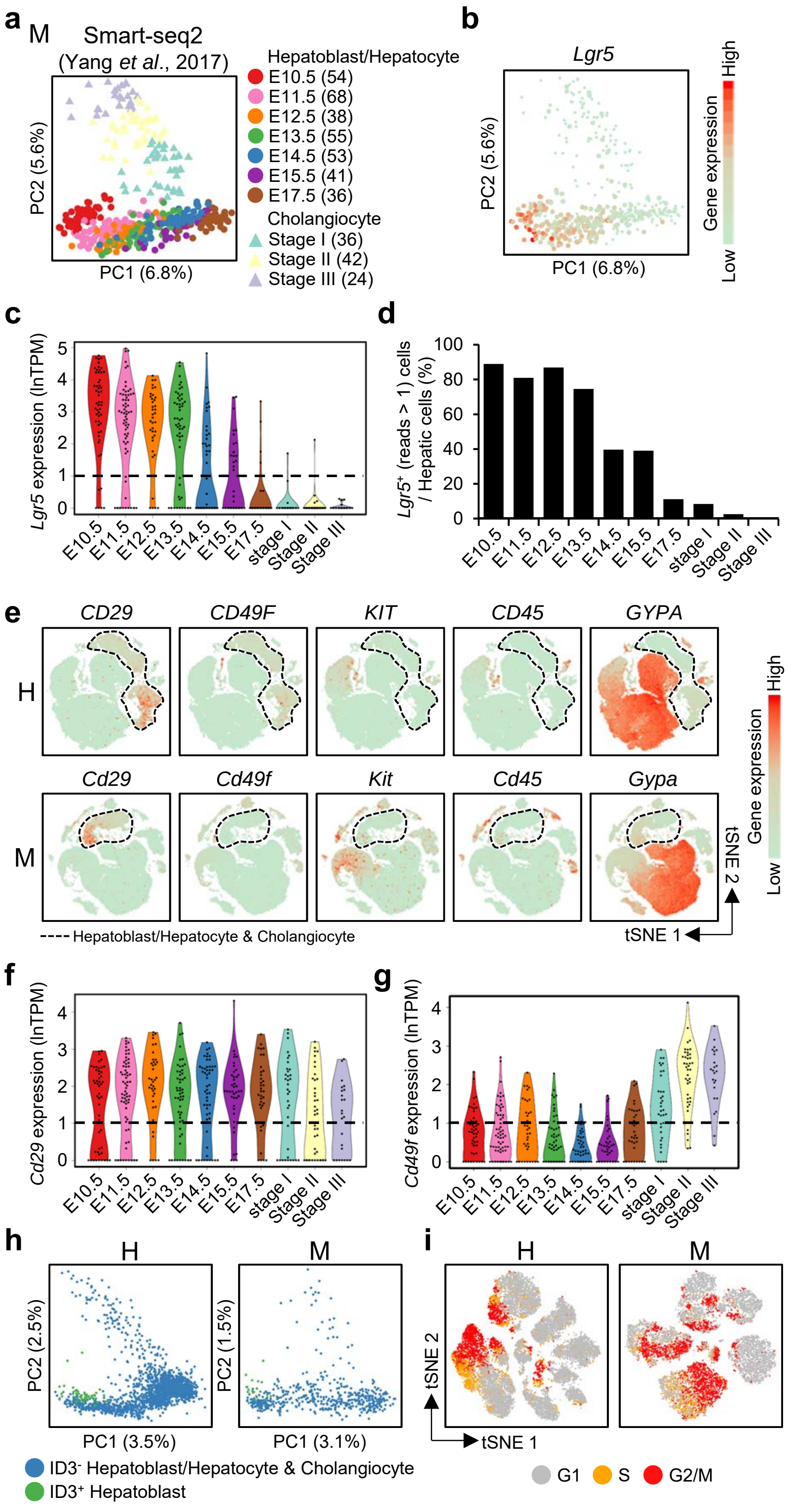

Supplementary information, Fig. S5 Expressions of fetal liver progenitor markers.

a PCA plot of Smart-seq2 datasets showing the classifications and stages of hepatoblasts/hepatocytes and cholangiocytes[5]. **b** PCA plot showing *Lgr5* expression in the hepatoblasts/hepatocytes and cholangiocytes. **c** Violin plots showing the expression levels of *Lgr5* in the hepatoblasts/hepatocytes and cholangiocytes. Each dot represents a single cell. The dashed line indicates the threshold of *Lgr5* expression. **d** The proportion of $Lgr5^{+}$ cells in the hepatoblasts/hepatocytes and cholangiocytes based on the Smart-seq2 analysis. **e** t-SNE plots showing the expression patterns of several genes. Hepatoblasts/hepatocytes and cholangiocytes are enclosed in the dashed lines. **f, g** Violin plots showing the expression levels of *Cd29* (**f**) and *Cd49f* (**g**) in the hepatoblasts/hepatocytes and cholangiocytes based on the Smart-seq2 analysis. Each dot represents a single cell. The dashed lines indicate the threshold of *Cd29* and *Cd49f* expression. **h** PCA plots showing the distribution of $ID3^{+}$ hepatoblasts in the hepatoblast differentiation pathways of humans (H) and mice (M). **i** Identification of cell cycle phases in hepatobiliary cells from human (H) and mouse (M) fetal livers.
