# Supplementary material for: Comparative analysis of cell lineage differentiation during hepatogenesis in humans and mice at the single-cell transcriptome level
Source: Cell Res. 2020 Jul 20;30(12):1109–26. doi: 10.1038/s41422-020-0378-6 (PMC7784864; doi:10.1038/s41422-020-0378-6)
Supplement: Supplementary file 6 — Supplementary information, Figure S6 [file 41422_2020_378_MOESM6_ESM.pdf]

Wang/Xu Figure S6

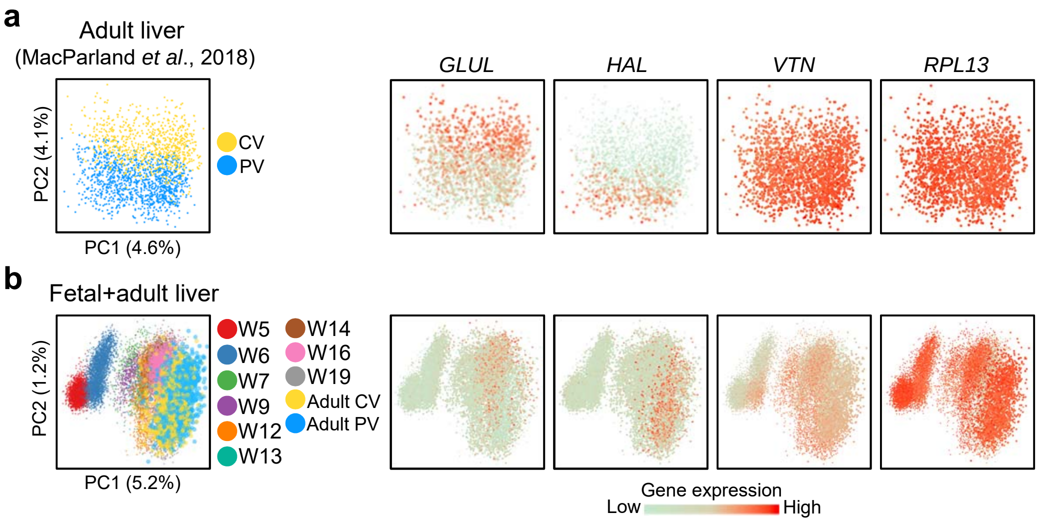

Supplementary information, Fig. S6 Analysis of heterogeneity of human hepatocytes. a PCA plots showing zonation and zonation-related genes in previously described adult human hepatocytes.[2] **b** Projection of adult human hepatocytes onto the PCA plot of human quiescent hepatoblasts/hepatocytes.
