# Supplementary material for: Comparative analysis of cell lineage differentiation during hepatogenesis in humans and mice at the single-cell transcriptome level
Source: Cell Res. 2020 Jul 20;30(12):1109–26. doi: 10.1038/s41422-020-0378-6 (PMC7784864; doi:10.1038/s41422-020-0378-6)
Supplement: Supplementary file 7 — Supplementary information, Figure S7 [file 41422_2020_378_MOESM7_ESM.pdf]

Wang/Xu Figure S7

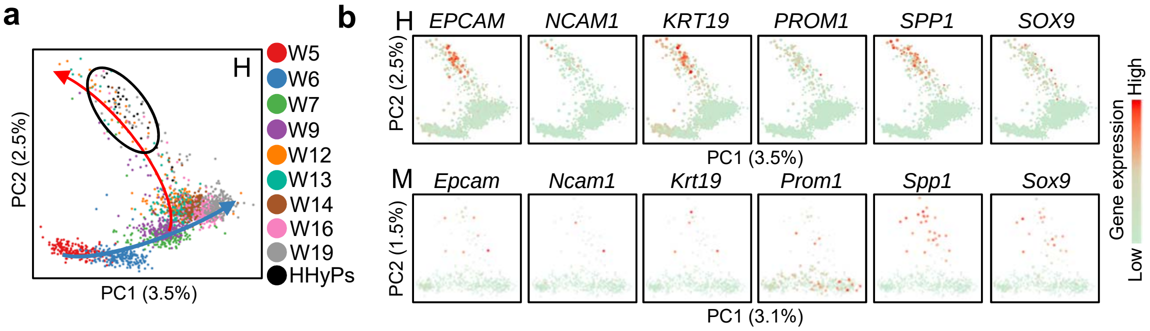

Supplementary information, Fig. S7 Analysis of HHyPs in the human hepatoblast differentiation pathway. **a** Projection of human HHyPs (black circle) onto the developmental pathway of human hepatoblasts. Arrows indicate the directions of hepatocyte (blue) and cholangiocyte (red) differentiation. **b** PCA plots showing the expression levels of HHyP-related markers in human (H) hepatobiliary cells, HHyPs, and mouse (M) hepatobiliary cells.
