# Supplementary material for: Comparative analysis of cell lineage differentiation during hepatogenesis in humans and mice at the single-cell transcriptome level
Source: Cell Res. 2020 Jul 20;30(12):1109–26. doi: 10.1038/s41422-020-0378-6 (PMC7784864; doi:10.1038/s41422-020-0378-6)
Supplement: Supplementary file 8 — Supplementary information, Figure S8 [file 41422_2020_378_MOESM8_ESM.pdf]

Wang/Xu Figure S8

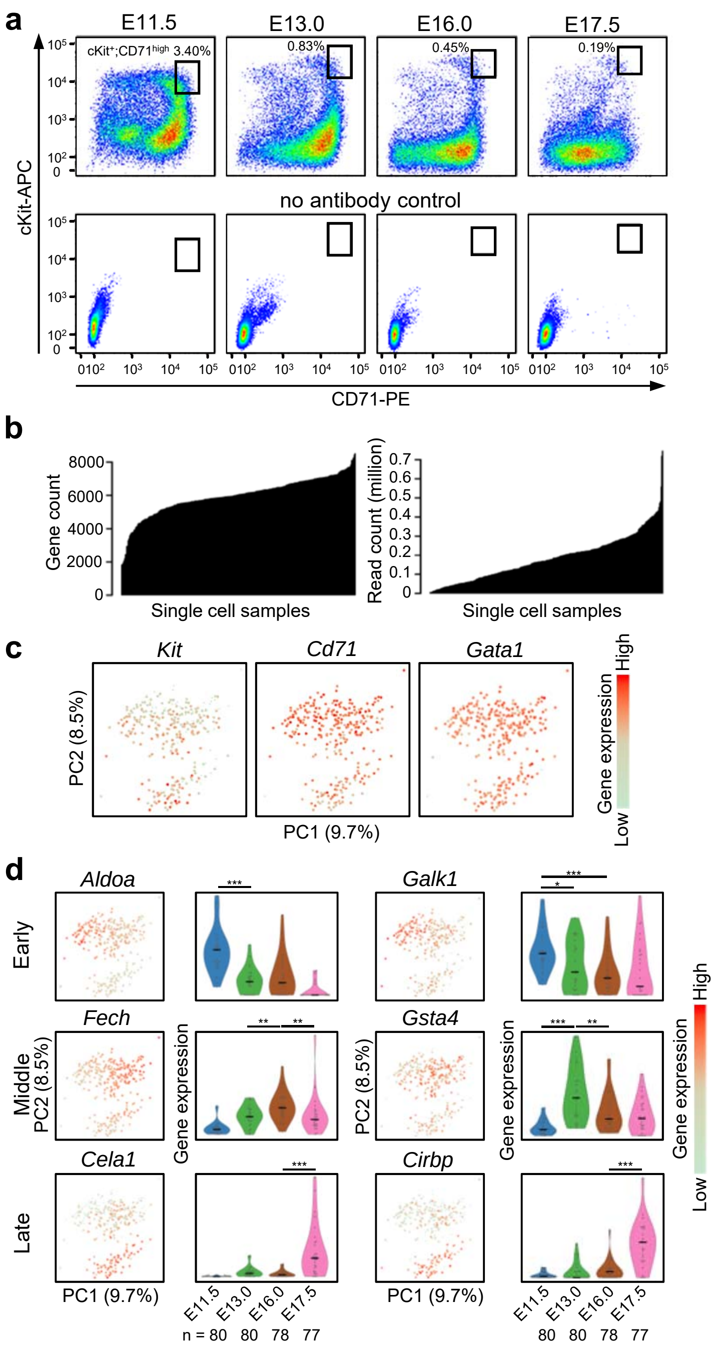

Supplementary information, Fig. S8 Isolation of mouse erythroid progenitors from fetal livers. a FACS plots showing the gating of $cKit^{+}CD71^{high}$ cells in E11.5-17.5 mouse whole blood cells. **b** Bar plots showing detected genes (left) and read counts (right) for mSTRT-seq data from mouse $cKit^{+}CD71^{high}$ cells. **c** PCA plots showing the expression levels of marker genes of erythroid progenitors in mouse $cKit^{+}CD71^{high}$ cells. **d** PCA plots showing the expression levels of marker genes in mouse $cKit^{+}CD71^{high}$ cells. Violin plots showing the expression levels of marker genes of E11.5-17.5 mouse $cKit^{+}CD71^{high}$ cells validated by single-cell RT-qPCR. The y-axis represents the relative expression values normalized to *Gapdh* expression. Each dot represents a single cell. The black line within each violin plot indicates the median of the expression levels. n, number of single cells. *$P < 0.05$; **$P < 0.01$; ***$P < 0.001$, Wilcoxon test.
