# Supplementary material for: Comparative analysis of cell lineage differentiation during hepatogenesis in humans and mice at the single-cell transcriptome level
Source: Cell Res. 2020 Jul 20;30(12):1109–26. doi: 10.1038/s41422-020-0378-6 (PMC7784864; doi:10.1038/s41422-020-0378-6)
Supplement: Supplementary file 9 — Supplementary information, Figure S9 [file 41422_2020_378_MOESM9_ESM.pdf]

Wang/Xu Figure S9

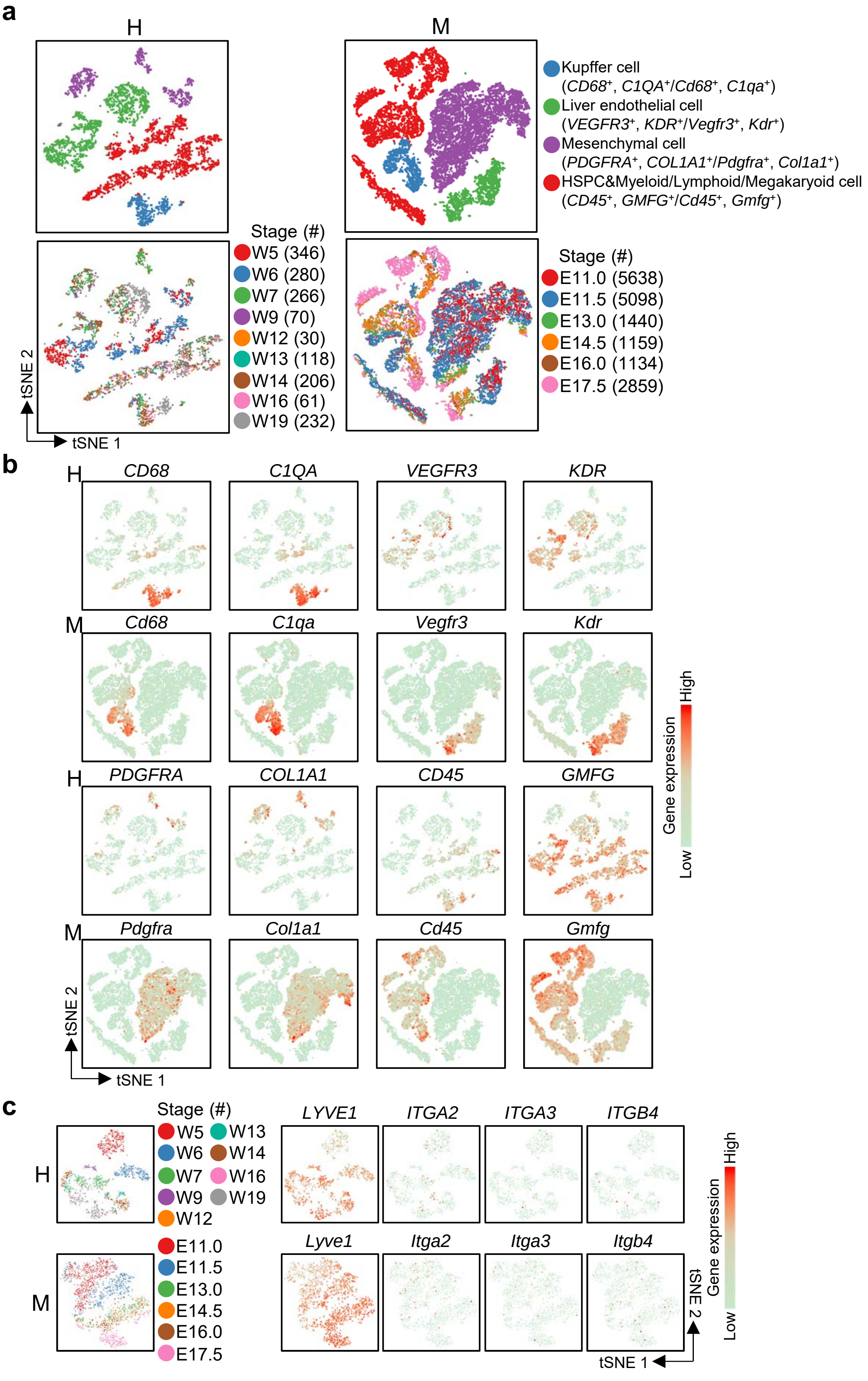

Supplementary information, Fig. S9 Analysis of mesoderm-derived non-erythroid cells. a t-SNE plots showing the classification (upper) and developmental stages (lower) of mesoderm-derived non-erythroid cells in human (H) and mouse (M) fetal livers. **b** t-SNE plots showing the expression levels of marker genes in human (H) and mouse (M) mesoderm-derived non-erythroid cells. **c** Left: t-SNE plots showing the classifications of liver endothelial cells in the human (H) and mouse (M) fetal liver. Right: t-SNE plots showing the expression levels of marker genes in human (H) and mouse (M) liver endothelial cells.
